# Dynamic methylation of histone H3K18 in differentiating *Theileria* parasites

**DOI:** 10.1038/s41467-021-23477-2

**Published:** 2021-05-28

**Authors:** Kevin Cheeseman, Guillaume Jannot, Nelly Lourenço, Marie Villares, Jérémy Berthelet, Teresa Calegari-Silva, Juliette Hamroune, Franck Letourneur, Fernando Rodrigues-Lima, Jonathan B. Weitzman

**Affiliations:** 1 Université de Paris, Epigenetics and Cell Fate, CNRS, Paris, France; 2 Université de Paris, Functional and Adaptive Biology, CNRS, Paris, France; 3 Université de Paris, Institut Cochin, Inserm, CNRS, Paris, France

**Keywords:** Epigenetics, Parasitology

## Abstract

Lysine methylation on histone tails impacts genome regulation and cell fate determination in many developmental processes. Apicomplexa intracellular parasites cause major diseases and they have developed complex life cycles with fine-tuned differentiation events. Yet, apicomplexa genomes have few transcription factors and little is known about their epigenetic control systems. Tick-borne *Theileria* apicomplexa species have relatively small, compact genomes and a remarkable ability to transform leucocytes in their bovine hosts. Here we report enriched H3 lysine 18 monomethylation (H3K18me1) on the gene bodies of repressed genes in *Theileria* macroschizonts. Differentiation to merozoites (merogony) leads to decreased H3K18me1 in parasite nuclei. Pharmacological manipulation of H3K18 acetylation or methylation impacted parasite differentiation and expression of stage-specific genes. Finally, we identify a parasite SET-domain methyltransferase (TaSETup1) that can methylate H3K18 and represses gene expression. Thus, H3K18me1 emerges as an important epigenetic mark which controls gene expression and stage differentiation in *Theileria* parasites.

## Abstract (web summary)

Post-translational modifications (PTM) of histones are important for epigenetic regulation in mammals. Here, Cheeseman et al. report dynamic changes in H3K18 PTM during the differentiation stages of Theileria parasites and they identify a SET-domain methyltransferase (TaSETup1) as the relevant enzyme promoting this modification.

## Introduction

Our ability to control infectious diseases is improved by our understanding of their complex life cycles and the characterization of pathogen-specific mechanisms that can be targeted by drug strategies. Apicomplexa parasites are a major cause of disease in humans and domesticated animals across the world. For example, *Plasmodium* species cause malaria affecting millions of people each year, emphasizing the need for effective drugs targeting parasites^1,2^. *Cryptosporidium* is a major cause of diarrhoea in developing countries following infection from contaminated water supplies and there is currently no effective drug therapy^3,4^. *Theileria* species are bovine-specific pathogens that cause diseases with significant economic impact; Tropical Theileriosis kills over a million cattle per year and costs in the hundreds of millions of dollars. The development of new therapeutic strategies is challenging, as Apicomplexa are eukaryotic cells and share many metabolic pathways with their host animals^2^.

Of all the apicomplexa parasites, *Theileria* is the only eukaryote known to transform its host cell and constitutes a unique model system to explore parasite–host interactions and microbial tumorigenesis^5,6^. Two Theileria species, *T. parva and T. annulata* are bovine-specific pathogens that cause severe disease following tick transmission. Infection by these *Theileria* species causes a lymphoproliferative disease in cows with clinical features similar to some human leukaemias^5,7,8^. *T. annulata* infects mainly bovine B cells and macrophages, whereas *T. parva* infects bovine B and T lymphocytes. *Theileria*-infected cells are transformed and immortalized; they display uncontrolled proliferation in vitro, independent of exogenous growth factors, and increased ability to migrate and form metastases in immunodeficient mice. Interestingly, *Theileria*-dependent transformation is reversible; animals treated with the theilericidal drug Buparvaquone are cured in most cases. When *Theileria*-infected cells are treated in vitro with Buparvaquone, the intracellular parasite diminishes in the host leucocytes, which lose the transformed phenotype, but drug resistance in the field is an emerging concern for disease control. To achieve transformation, the parasite manipulates the host cell signalling pathways that control cell proliferation and survival^5,6,8^. Several host signaling pathways have been implicated in *Theilieria*-induced transformation including metabolic pathways^9,10^, c-Jun Nterminal Kinase (JNK) signaling^11^ and host nuclear factors, such as c-Myc, E2F and AP-1^12–15^. A parasite secreted factor, TaPin1, activates host signaling pathways leading to activation of oncogenic host c-Jun and metabolic gene expression^16–18^. While previous studies have focused on the host signaling pathways activated by intracellular parasites^6,15^, very little is known about the regulation of the parasite genome and the mechanisms that orchestrate parasite differentiation and its complex life cycle. A previous study monitored gene expression through merogony, the process of parasite differentiation from intracellular schizonts to infectious merozoites, and reported interesting changes in the levels of transcription factors of the AP2 family^19^. However, the relative paucity of transcription factors in *Theileria* genomes^20^ suggests that other mechanisms such as epigenetic pathways may also contribute to parasite differentiation.

Many diseases, especially cancer, are linked to epigenetic events that lead to changes in gene expression. Epigenetic changes associated with disease states include DNA methylation and histone modifications such as lysine methylation and acetylation^21,22^. Epigenetic enzymes have been causally linked to many diseases making them promising targets for drug interventions^23^. Recently novel drugs that inhibit methylation or deacetylation were developed and some obtained FDA approval to treat cancer. Notably, lysine methylation is emerging as a versatile and dynamic post-translational modification (PTMs) that contributes critically to cellular differentiation programmes^24^. The human genome encodes about 50 biochemically validated lysine methyltransferases (KMTs) that ‘write’ the methylation code and 20 lysine demethylases (KDMs) that act as ‘erasers’. Numerous reports of misregulation of KMTs and KDMs in cancer drove an intense search for specific small-molecular inhibitors^21^. Despite these advances, relatively little is known about the role of epigenetic proteins (methylation Writers or Erasers) in infectious diseases or in infection-induced cancers^25,26^. The posttranslational modification of lysine residues in the histone N-terminal tails plays an important role in regulating chromatin structure and gene expression in all eukaryotes^22^, but has not been previously studied in *Theileria* parasites. We hypothesized that epigenetic modifications, particularly lysine methylation of histone tails, could be a feature of parasite differentiation and that the characterization of parasite encoded epigenetic enzymes could be future drug targets for anti-parasite therapies. In this work we describe the role of methylation of histone H3K18 as an important gene regulatory event during the differentiation of *Theileria* parasites and identify the first parasite methyltransferase capable of methylating H3K18.

## Results

### Parasite histones are methylated at H3K18

**Fig. 1:**
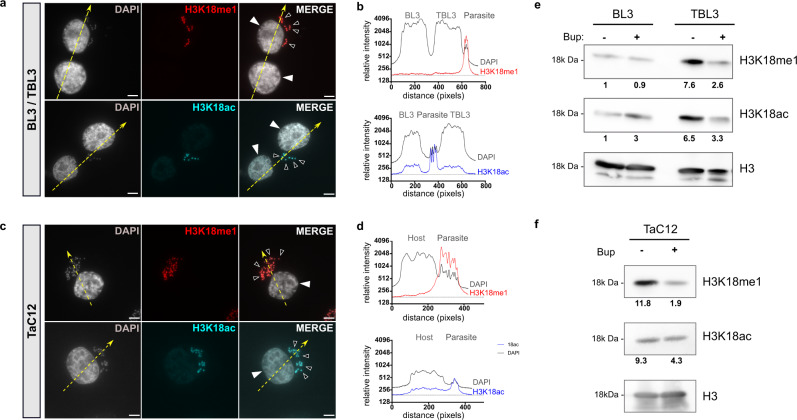
H3K18 methylation marks parasite nuclei. **a** Immunofluorescence analysis of BL3 (uninfected) mixed with TBL3 (infected) cells. Host and parasite nuclei are stained with DAPI (grey). Histone marks were detected with specific antibodies for H3K18me1 (red) or H3K18ac (cyan). Solid white arrowheads indicate the nuclei of the bovine cells and the smaller empty arrowheads point to parasite nuclei in TBL3 cells only. The yellow dotted arrows indicate the cross-section planes used for quantification. Scale bar = 5 μm. **b** Quantification of immunofluorescence intensity of H3K18me1 (red) and H3K18ac (blue) compared to DNA/DAPI (grey). The plot profiles are representative of the yellow cross-section lines. **c** Immunofluorescence staining for H3K18me1 (red) and H3K18ac (cyan) in parasite-infected macrophages (TaC12 cells). Host and parasite nuclei are stained with DAPI (grey). Leica microscope, ×100, Scale bar = 5 μm. Solid white arrowheads show the bovine host nuclei and empty arrowheads point to parasitic nuclei. The yellow dotted arrows indicate the cross-section planes used for quantification. **d** Quantification of immunofluorescence intensity of H3K18me1 (red) and H3K18ac (blue) compared to DNA (grey). The plot profiles are representative of the yellow cross-section lines. **e** Western blot analysis of BL3 and TBL3 cells (treated with the theilericidal drug Buparvaquone, Bup). H3 was used as a loading control. The bands were quantified and compared with uninfected BL3 cells. Results are representative of at least three independent experiments. **f** Western blot analysis of H3K18 modifications in infected TaC12 macrophages treated with or without Buparvaquone (Bup). The bands were quantified and compared with uninfected BL3 cells. Results are representative of at least three independent experiments. All these experiments were performed three times independently with similar results; representative experiments are shown. Full scans blot and immunofluorescence replicates are included in the Source Data file.

To initiate a study of epigenetic regulation in *Theileria* parasites, we examined parasite histones focusing on H3. Our analysis of the *T. annulata* genome revealed the presence of two genes encoding histone H3 (Supplementary Fig. 1). The sequences of the N-terminal tails, especially the Lysine residues, are particularly well-conserved in the H3 proteins from *T. annulata*, *T. parva* and mammals (Supplementary Fig. 1). We, therefore, examined histone modifications using a panel of commercial antibodies recognizing different modified lysine residues in H3 tails. Many of the antibodies we tested by immunofluorescence staining showed strong signals in both host and parasite nuclei; these included relatively well-studied marks such as H3K4me3 and H3K36me3 (see below). However, one modification caught our attention: antibodies recognizing mono-methylated H3K18 (H3K18me1) detected *T. annulata* parasite nuclei, but did not stain bovine host nuclei (Fig. 1a, b). We conducted a series of experiments to pursue the specificity of this initial observation. In contrast to H3K18me1, antibodies against acetylated H3K18 (H3K18ac) displayed strong immunofluorescence signals in both host and parasite nuclei in infected and non-infected bovine B cells (Fig. 1a, b). We observed similar parasite-specific staining for H3K18me1, but not for H3K18Ac, in *T. annulata*-infected macrophages TaC12 (Fig. 1c, d) and in lymphocytes infected with related *T. parva* parasites (Supplementary Fig. 2). Further control experiments with three independent antibodies demonstrated the specificity of the antibody for mono-methylated H3K18 residues, with no cross-reaction to other well-studied H3 lysine methylations (Supplementary Fig. 3). Notably, the sequence around the H3K18 residue is extremely similar in parasite or bovine histones (Supplementary Fig. 1). The difference in monoisotopic mass between the Serine-22 and Threonine-22 residues is virtually the same as a methyl group (14.01565): so mass spectrometry could not be used effectively to distinguish between parasite and bovine H3K18 modifications. We confirmed the presence of methylated and acetylated H3K18 in TBL3, TpMD409 and TaC12^27^ parasite-infected cells by immunoblot experiments (Fig. 1e, f and Supplementary Fig. 2b).

Treatment with the theilericidal drug Buparvaquone (Bup), which kills the parasites, reduced H3K18me1 levels in cell lines infected with *T. annulata* or *T. parva* (Fig. 1e, f and Supplementary Fig. 2b). From these experiments, and careful controls, we conclude that H3K18 mono-methylation appears to be a feature of *Theileria* parasite schizonts.

### H3K18me1 is enriched on the gene bodies of repressed genes in Theileria schizonts

**Fig. 2:**
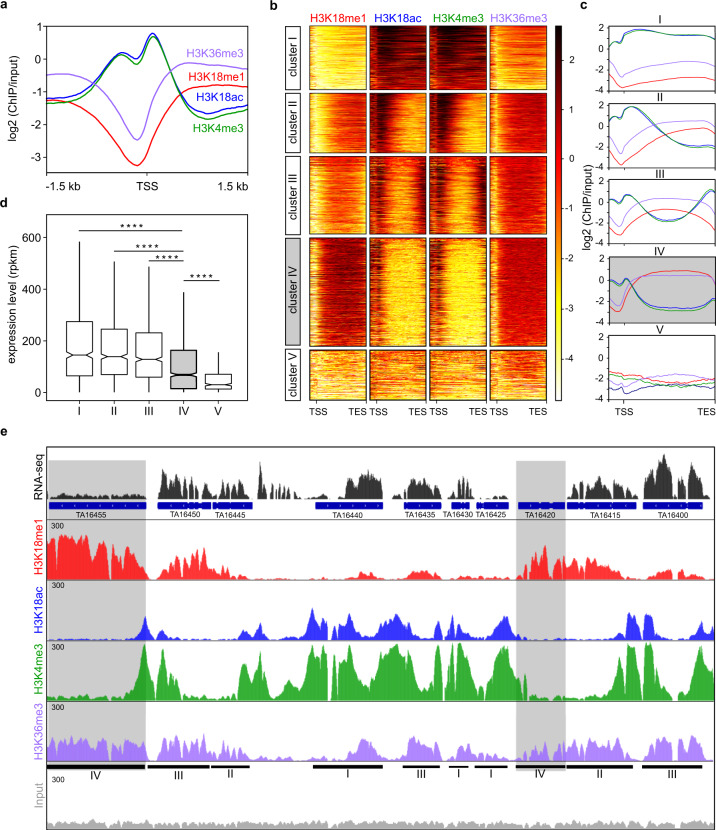
Mono-methylation of H3K18 is distributed on the gene bodies of repressed genes. **a** Average occupancy profiles for H3K4me3 (green), H3K18ac (blue), H3K18me1 (red) and H3K36me3 (purple) around transcriptional start sites (TSS) of all *T. annulata* genes. *X*-axis: genomic coordinates ± 1.5 kb. *Y*-axis: log2 (ChIP/Input). **b** Heatmap of occupancy across all *Theileria* genes starting from 500 bp before the TSS to the end of the gene (TES), normalised by length (normalised to input read coverage). Clustering is the result of a 5 k-means clustering on H3K18me1. The H3K18me1-enriched Cluster IV is highlighted in light grey. **c** Average occupancy profiles per cluster for H3K4me3 (green), H3K18ac (blue), H3K18me1 (red) and H3K36me3 (purple) starting from 500 bp before the TSS to the TES, normalised by length. Cluster IV is highlighted in grey. **d** Boxplot of gene expression (RPKM) per cluster as defined by the 5 k-means clusterings on H3K18me1. Pairwise-Wilcoxon statistical testing is indicated. Custer IV is highlighted in grey. Statistical test Dunnett’s multiple comparison test: ****p < 0.0001. A two-sided pairwise Wilcoxon test was performed. Values: ns: *p* > 0.05,*p < 0.05, **p < 0.01,***p < 0.001,****p < 0.0001. Minimum and maximum are represented by upper and lower Whiskers, respectively. Interquartile representation includes first and third quartile, centre representing median, test was performed on three RNA-Seq replicates. **e** Selected genome region depicting epigenetic profiles and gene expression for *Theileria* genes belonging to the four clusters (indicated below). Two genes from cluster IV are highlighted in grey (*TA16455*, *TA16420*). H3K4me3 (green), H3K18ac (blue), H3K18me1 (red) and H3K36me3 (purple), Input (grey). The region ranges from gene *TA16455* to *TA16400*, chr1: 2346753–2365563 (*T. annulata* Ankara strain, accessible at https://www.piroplasmadb.org).

Acetylation of H3K18 is a well-studied epigenetic mark linked to gene activation^28^, whereas no studies have focused on the functional role of H3K18 methylation in gene regulation. Indeed, no specific gene regulatory functions have been ascribed to H3K18me1. To investigate H3K18 modifications on the *Theileria* genome, we performed chromatin immunoprecipitation followed by sequencing (ChIP-Seq, performed in duplicates, which we subsequently merged upon good replicate correlations, see Supplementary Fig. 5, to generate the figures shown here) with several antibodies and parallel RNASeq transcriptome analysis. Our dual-sequencing in infected TBL3 cells, followed by reading mapping and bioinformatics analysis, allowed us to simultaneously map epigenomic features on both the parasite and the bovine host genomes. Our analysis of the parasite epigenome revealed some patterns previously described on mammalian genomes; namely, promoter regions around the transcriptional start site (TSS) were enriched for ‘activating’ epigenetic marks such as H3K4me3 or H3K18ac in the parasite genome (Fig. 2a). In contrast, H3K18me1 levels were lowest at the TSS and enriched on the gene bodies (Fig. 2a). Analysis of the bovine genome in the same ChIP-Seq experiments showed enrichment for H3K4me3 and H3K18ac on promoter regions, but no H3K18me1 enrichment on gene bodies, consistent with the parasite-specific immunofluorescence staining results (Supplementary Fig. 4a, b). Gene-body enrichment on actively transcribed genes is a feature of H3K36me3 methylation in mammals^29^. We observed gene-body enrichment for H3K36me3 on genes in the parasite genome (Fig. 2e and Supplementary Fig. 4c). We observed a strong correlation between gene promoter H3K4me3 and H3K18ac, and a weaker correlation between H3K18me1 and H3K36me3 in the parasite genome (Supplementary Fig. 5a–c). The H3K18me1 and H3K36me3 profiles are, however, quite distinct; a differential peak-calling approach on these two modifications revealed differences in the number of peaks (1537 for H3K18me1, 3122 for H3K36me3). We performed k-means clustering analysis of the H3K18me1 profiles on the *T. annulata* parasite genome and defined five clusters (Fig. 2b). Cluster I and Cluster V had the lowest levels of H3K18me1, whereas Cluster IV genes (1103 genes) were enriched for gene-body H3K18me1 (Fig. 2c). In contrast, H3K36me3 levels were relatively high across all clusters, except Cluster V. Comparison with our RNA-Seq data revealed that Cluster IV genes were characterized by lower gene expression compared to Clusters I–III (Fig. 2d, e). Notably, Cluster IV genes were particularly enriched for gene-body H3K18me1 which appeared mutually exclusive with H3K18ac and correlated with exonic sequences (Fig. 2e and Supplementary Fig. 5d) (*p* < 0.0001). The genes enriched for H3K18me1 do not appear to cluster on the genome or to the group in particular chromosomal locations (Supplementary Fig. 6). Methylation of parasite H3K36 has been linked to repression of *var* genes in *Plasmodium* parasites^30^, but we did not observe regions of strikingly high H3K36me3 enrichment in the *Theileria* genome (Supplementary Fig. 6). Hence, gene-body enrichment of the H3K18me1 modification appears to be correlated with repression of a large number of genes in *T. annulata* schizonts.

### Parasite differentiation is associated with dynamic changes in H3K18 modifications

**Fig. 3:**
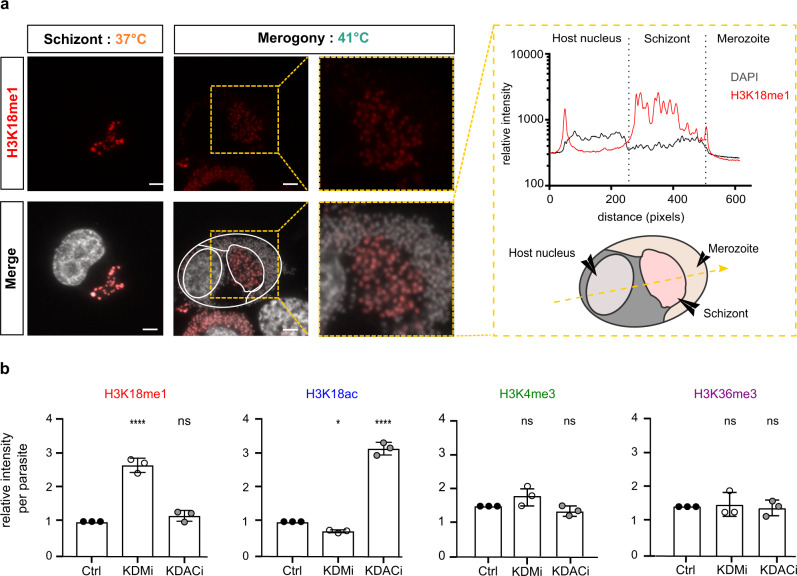
H3K18 methylation is dynamic across stage transitions. **a** Differentiation to merozoites led to a decrease of H3K18me1 in parasite nuclei Immunofluorescence analysis of *T. annulata* infected macrophages TaC12, at 37 °C (macroschizont) and after merogony induction for 8 days at 41 °C. Host and parasite nuclei were stained with DAPI (grey) and with a specific antibody for H3K18me1 (red). The yellow box shows a zoom of merogony. Leica microscope, ×100, Scale bar = 5 μm. The right panel shows quantification of immunofluorescence intensity of H3K18me1 (red) compared to DNA (grey), along the yellow cross-section line, showing reduced staining in merozoites. This experiment was performed three times independently with similar results. Immunofluorescence replicates are included in the Source Data file. **b** Changes in the level of Histone methylation and acetylation upon treatment with inhibitors of epigenetic enzymes. Fluorescence intensity quantification of the parasite histone marks H3K18me1, H3K18ac, H3K4me3 or H3K36me3 in TaC12 cells treated with either KDMi (histone demethylase inhibitor) or KDACi (histone deacetylase inhibitor). For all experiments *n* = 3 biologically independent experiments that represent the mean of intensity of 50 cells per condition for each replicate. Error bars represent the mean value ± SD. Statistical Dunnett test multiple comparisons is two-sided with ns (not statistically significant) of K18m *p* = 0.2979/ns of K18ac *p* = 0.2435/ns of K4 *p* = 0.5060/ns of K36 *p* > 0.9999/****p = 0.0001.

**Fig. 4:**
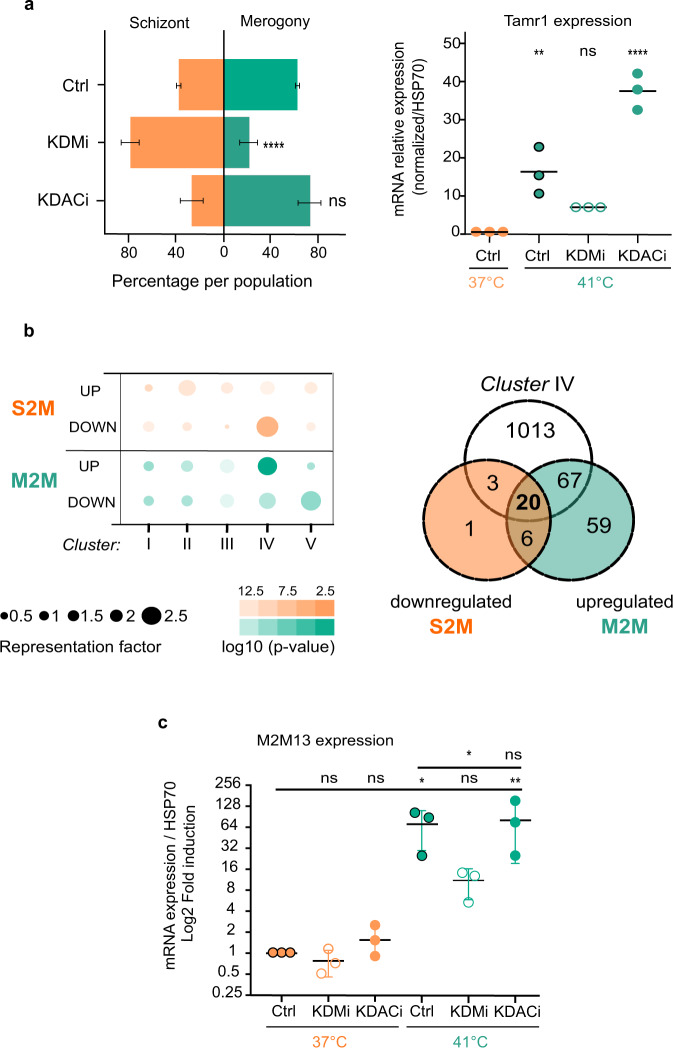
Pharmacological treatments impact parasite differentiation. **a** KDMi and KDACi treatment impacted merogony induction in infected cells. Quantification of cells at macroschizont (orange) or merogony (green) parasite stages after 8 days cultured at 41 °C treated with inhibitors, biologically independent experiments in triplicate, mean of the cells in schizont or merogony stage for each replicate. The right panels shows the expression levels of the parasite gene *Tamr1* (merogony marker) after 8 days at 41 °C treated with inhibitors. Levels at 41 °C were compared to 37 °C schizont controls *n* = 3 biologically independent experiments represent qPCR values of TamR1 expression normalized by Hsp70 for three different induction of merogony treated or not. Error bars represent mean values ± SD. Statistical test Dunnett’s multiple comparison and two-sided test: ns = not statistically significant, *p* = 0.1195; ****p = 0.0001; **p = 0.0031. **b** Cluster IV genes are over-represented in differentially expressed genes in sporozoite-tomacroschizont (S2M down-regulated) and macroschizont-to-merozoite (M2M up-regulated) transitions. Dot chart of the representation factor and *p*-value of significant overlap between differentially expressed genes derived from a published microarray^19^ and our H3K18me clusters. Dot colour transparency is plotted as *p*-value and dot size represents the representation factor. The right panel shows Venn diagram indicating the numbers of genes overlapping between cluster IV, genes upregulated in the M2M transition and genes downregulated in the S2M transition. **c** Merogony induction increased the expression of the *M2M13* gene (Cluster IV) that is upregulated during macroschizont to merozoite transition. The *M2M13* gene was affected by the epigenetic inhibitors KDMi and KDACi. RT-qPCR analysis of *M2M13* gene in infected macrophages, at 37 °C or at 41 °C for 8 days, after treatment with inhibitors, showed that the expression level of *M2M13* was significantly reduced by KDMi treatment. Levels at 41 °C were compared to 37 °C schizont controls or untreated merogony (*n* = 3 biologically independent experiments, mRNA expression of *M2M13* normalized to *Hsp70*, for three different experiments on schizont treated or not and for three inductions of merogony treated or not). Error bars represent mean values ± SD. Statistical test Dunnett’s multiple comparisons two-sided: ns = not statistically significant > 0.99; **p < 0.005; *p = 0.0312.

In other apicomplexan species, dynamic changes in epigenetic histone modifications accompany changes in gene expression throughout the parasite life cycle^25,26,31^. Important work from the Shiels laboratory began to define changes in gene expression in *Theileria* parasites during stage differentiation and implied roles for the ApiAP2 factors^19^. Little is known about the role of epigenetic mechanisms in regulating gene expression during *Theileria* differentiation. To investigate the dynamics of H3K18 modifications in *Theileria* parasites, we induced parasite differentiation from schizonts to merozoites (‘merogony’), by the sustained culture of infected macrophages at elevated temperatures^32^. The induction of merogony was accompanied by a reduction in H3K18me1 staining in some parasite nuclei (Fig. 3a). After 8 days in the culture at 41 °C, we observed large patches of parasite nuclei with reduced H3K18me1 staining, despite robust H3K4me3 staining across all nuclei (Supplementary Figs. 7 and 8). This suggested that H3K18 methylation dynamics might contribute to parasite differentiation. Unfortunately, there are currently no genetic techniques available to modify the *Theileria* genome, so we are limited to pharmacological intervention. To test H3K18 dynamics experimentally, we treated cells with inhibitors of lysine demethylases (KDMi) or deacetylases (KDACi), some of the many drugs being developed to inhibit epigenetic enzymes^23^. Treatment with a demethylase inhibitor led to a 3-fold increase in parasite H3K18me1 levels and a modest decrease in H3K18ac (Fig. 3b). Conversely, treatment with an acetylase inhibitor robustly increased H3K18ac staining in parasites (Fig. 3b). Importantly, treatment with KDMi or KDACi did not affect the levels of parasite staining with antibodies against H3K4me3 or H3K36me3 (Fig. 3b), demonstrating that the inhibitors do not have a general broad effect on histone lysine methylation. With these pharmacological tools in hand, we sought to test whether these drug-induced effects on H3K18 methylation and acetylation could influence parasite differentiation. We speculate that increased H3K18 methylation might block differentiation, or increased acetylation might favour differentiation. We observed that KDMi treatment significantly reduced the extent of merogony and reduced the expression of the differentiation marker *TamR1*^32^ (Fig. 4a). Conversely, treatment with KDACi enhanced *TamR1* expression, with a relatively mild impact on differentiation (Fig. 4a). These results suggested that dynamic changes in the H3K18 methylation/acetylation balance could affect parasite stage differentiation.

**Fig. 5:**
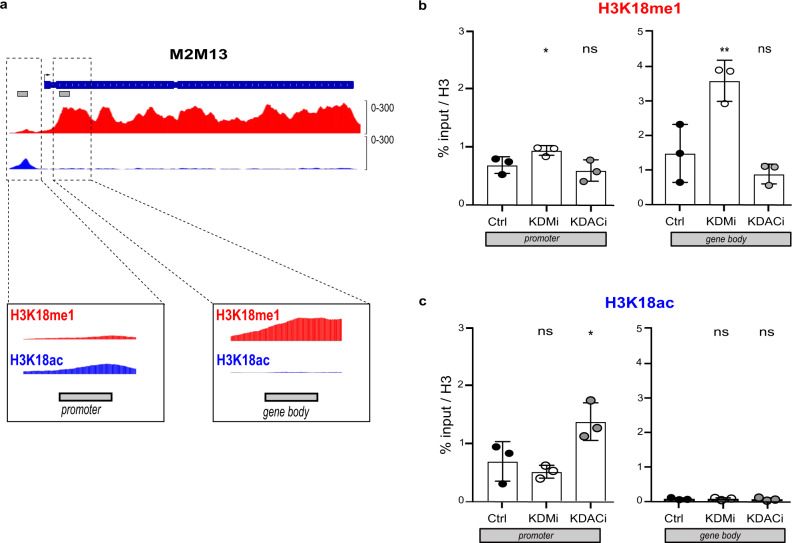
Chromatin analysis of a differentiation gene. **a** Representation of the chromatin status of H3K18 at the *M2M13* locus. Chromatin immunoprecipitation ChIP-Seq analysis of the *M2M13* gene in schizont conditions (37 °C) showing H3K18Ac enrichment (blue) on the *M2M13* promoter region and H3K18me enrichment (red) on the gene body. **b** Treatment with the KDMi or KDACi inhibitors impacted the presence of H3K18me1 histone marks on the *M2M13* gene. Quantification of the H3K18me1 mark by ChIP-PCR on the *M2M13* promoter or gene-body, after treatment with epigenetic inhibitors. **c** Quantification of H3K18ac mark on the M2M13 gene and promoter following inhibitor treatment. For all experiments *n* = 3 biologically independent experiments of ChIP qPCR. Error bars represent mean values ± SD. Statistical test Dunnett’s multiple comparison test: ns = not statistically significant *p* = 0.2979; **p = 0.047; *p = 0.0737.

Previous microarray analysis identified sets of genes associated with life-stage transitions in *T. annulata*^19^. Comparison with these data revealed that Cluster IV genes, which are enriched for genebody H3K18me1, are over-represented in differentially expressed genes linked to the sporozoite-tomacroschizont and macroschizont-to-merozoite stage transitions (Fig. 4b and Supplementary Fig. 8). Notably, nine genes encoding *Theileria* ApiAP2 genes that could be stage-transition determinants^19^ are also in the list of cluster IV genes (Supplementary Table 3). We analysed the overlap between these datasets, focusing on Cluster IV genes and correlations with genes reported to be downregulated in the sporozoite-to-macroschizont (S2M) transition and upregulated in macroschizont-to-merozoite (M2M) transition. We identified 20 candidate genes (that belong to both M2M_up and S2M_down gene sets, which we termed M2M1-M2M20) that are candidates for genes that are repressed by high H3K18me1 in macroschizonts and upregulated upon differentiation (Fig. 4b and Supplementary Table 1). We also performed a Blast2GO re-annotation of the additional 67 genes that are in the M2M_up group and contained in Cluster IV (Supplementary Table 2). Notably, the 87 genes on these lists (which are candidates for increased expression during the transition from macroschizont to merozoite) include several genes with FAINT domains and signal peptides or transmembrane domains that could be involved in host interactions. It is particularly noteworthy that three proteins (TA05870, TA16660 and TA19445) are homologues of rhoptry proteins characterized in *Plasmodium* or *Toxoplasma* as functionally linked to invasion and egress (Supplementary Tables 1 and 2). Gene expression analysis showed that 5 of these 20 genes are indeed significantly induced (over 10-fold) in our hands upon merogony culture conditions (Supplementary Fig. 9a). In contrast, control genes (e.g. parasite *TaJmjC1*) or bovine genes encoding actin or Hsp70 did not significantly vary upon merogony induction (Supplementary Fig. 9b). We tested whether the drugs that affected H3K18 methylation and acetylation levels could impact the expression of the M2M genes and noted a significant tendency to be induced by treatment with KDACi (see examples in Supplementary Fig. 9c). We chose to study the *M2M13* gene (*TA16660* encoding a homologue of rhoptry neck proteins) as a striking example (Fig. 4c). The *M2M13* gene was induced 60-fold upon merogony differentiation compared to cells grown at 37 °C (Fig. 4c). The temperature-induced expression of *M2M13* was blunted by treatment with KDMi at 41 °C (Fig. 4c). To test whether these changes in expression were linked to changes in epigenetic effects and chromatin marks, we performed ChIP analysis of the *M2M13* gene locus (Fig. 5a). The M2M13 gene has a peak of H3K18Ac in the promoter region and enriched H3K18me1 across the gene body (Fig. 5a). Treatment with KDMi enhanced H3K18me1 on the *M2M13* gene body and promoter (Fig. 5b), whereas the KDACi treatment led to increased H3K18ac on the promoter region (Fig. 5c). This pharmacological intervention changed H3K18 modifications that correlated with gene expression and differentiation (Fig. 4e). Thus, the balance between H3K18me1 on gene bodies and H3K18ac on gene promoters could determine the expression of key genes associated with differentiation stage transitions in *Theileria* parasites.

### The Theileria SET-domain methyltransferase TaSETup1 can methylate H3K18

**Fig. 6:**
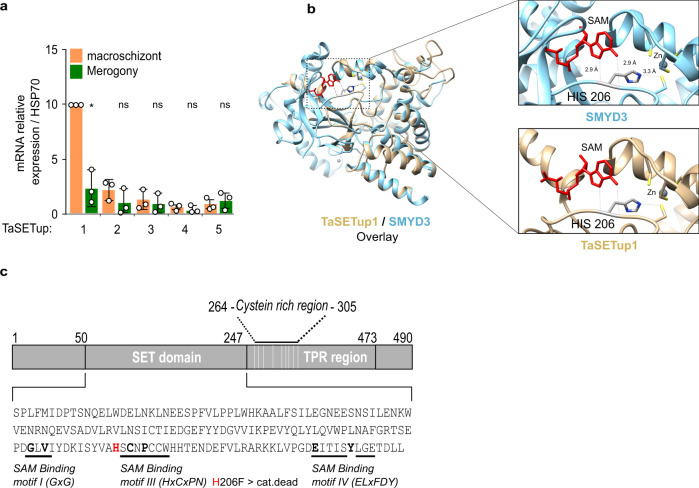
The *Theileria* methyltransferase TaSETup1. **a** Analysis of expression (RT-qPCR) the *TaSETup* genes in macroschizont or merogony culture conditions (37 or 41 °C, respectively). Results were normalized to *TaSETup1* at 37 °C and represent three independent experiments (*n* = 3). Statistical test Dunnett’s multiple comparison two-sided tests showing changes upon merogony: ns=not statistically significant > 0.6389; *p = 0.0488. **b** Comparison of TaSETup1 predicted structure (beige) with the crystal structure of the human SMYD3 protein (blue, PDB ID: 6IJL), with zoom window of the active site highlighting TaSETup1 H206 residue (red). **c** Schematic of the TaSETup1 protein, highlighting the SAM binding regions and the H206 residue (red).

**Fig. 7:**
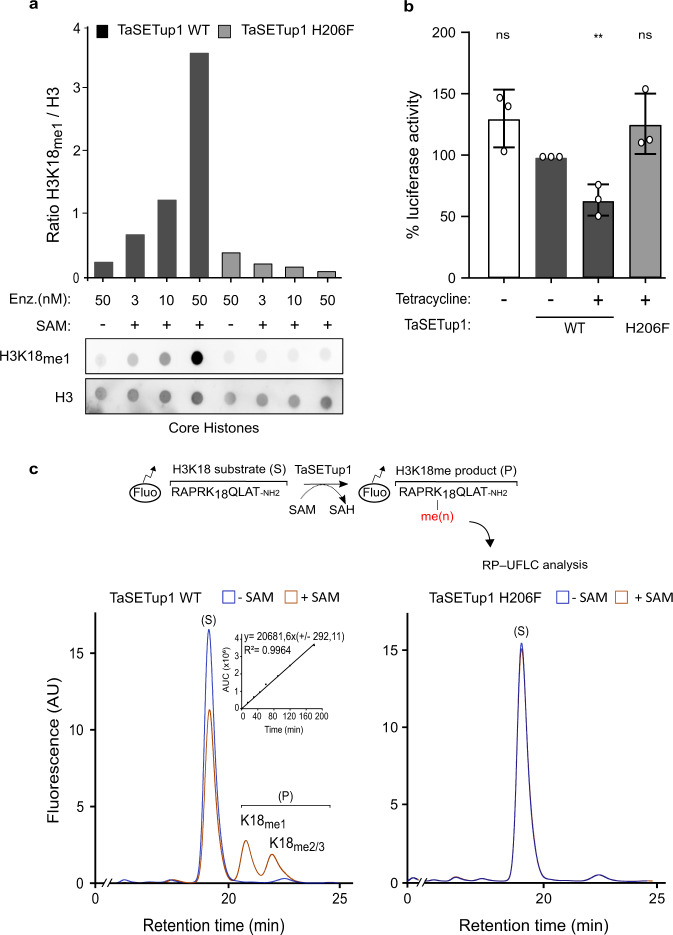
The *Theileria* TaSETup1 can methylate H3K18 and repress gene expression. **a** Methyltransferase assay of recombinant TaSETup1 (wild-type WT or mutant H206F) incubated with or without SAM and core histones followed by immuno-dot-blot detection with H3K18me1 antibody. The quantification results were normalized to H3. This result is representative of three independent experiments showing similar outcomes. **b** Assay of TaSETup1 methyltransferase activity by quantification of fluorescent H3K18 peptide (substrate S and methylated product P). Chromatograms show mono-methylated (K18me1) and di/tri methylated (K18me2/3) product in presence or absence of SAM for WT and mutant enzymes. Inset: Integration of the peak (AUC area under the curve). **c** Recruitment of GAL4-TaSetup1 (but not the GAL-TaSETip1 H206F mutant) caused repression of the *Luciferase* transgene in T-Rex cells in the presence Tetracycline (48 h). The % Luciferase activity represents the mean of *n* = 3 biologically independent experiments. Error bars represents mean values ± SD.

The methylation of lysine residues is typically performed by methyltransferases with a characteristic SET domain^33^. We mined the *T. annulata* genome^20^ for genes encoding SET-domain containing proteins and identified five candidates (termed here TaSETup1–TaSETup5) (Supplementary Fig. 10 and Supplementary Table 4). Our analysis of expression levels showed that only one of these, the uncharacterized protein TaSETup1, was expressed in macroschizonts and significantly silenced upon merogony differentiation (Fig. 6a). We also identified two potential demethylases with JMJ domains, one of which was induced upon merogony (Supplementary Fig. 10a, b). These observations are consistent with a methylation shift during differentiation. We performed phylogenetic analysis of the parasite putative methyltransferases and found that TaSETup1 resembles a family of mammalian methyltransferases called SMYD^34–36^ proteins that have been implicated in differentiation and cancer (Supplementary Fig. 11). We used knowledge of SMYD protein structure and function to predict residues whose mutation would lead to a catalytically inactive protein (Fig. 6b, c)^34^. Modelling on the known structure of the mammalian SMYD3^37^ protein predicted that mutation of the histidine residue at position 206 should inhibit activity. We performed a highly sensitive, in vitro SAM methyltransferase assays^38^ with recombinant TaSETup1 or mutant H206F. Recombinant TaSETup1 could methylate peptides corresponding to H3K18 residues to produce H3K18me1 and H3K18me2/3, whereas the H206F mutant was inactive (Fig. 7a, b). Furthermore, the recombinant TaSETup1 protein methylated core histones (Fig. 7a) as well as recombinant histone H3 or human nucleosomes (Supplementary Fig. 12a–c). We compared the activity of TaSETup1 with SMYD3, the most similar mammalian SET-domain enzyme. Recombinant TaSETup1 could methylate H3K18me1 on recombinant H3, polynucleosomes or core histone isolated from HEK cells (Supplementary Fig. 13a). In contrast, the related SMYD3 enzyme failed to methylate any of these substrates on K18. But the SMYD3 protein could methylate its preferred substrate MAP3K2^39^ in an in vitro SAM assay (Supplementary Fig. 13b), whereas TaSETup1 could not. Finally, we performed mass spectrometry analysis of histone peptides methylated by the recombinant TaSETup1 enzyme and demonstrated that the parasite enzyme catalyzed monomethylation of the lysine residue corresponding to H3K18 (Supplementary Fig 14). Thus, these two highly related methyltransferases exhibit a degree of specificity for different target substrates. No human methyltransferase has been demonstrated to methylate H3K18.

The genetic tools do not currently exist to test TaSETup1 function in live parasites by mutational analysis or knockdown strategies. We attempted to make antibodies that specifically recognized the TaSETup1 protein. Although we obtained antisera that recognized the immunizing antigen and recombinant TaSETup1 protein, these antibodies failed to produce satisfying results in immunoblot or immunofluorescence analysis. To circumvent these constraints, we tested the functional capacity of TaSETup1 to repress gene expression in a heterologous system as H3K18me1 was enriched on the gene bodies of repressed genes. We fused the parasite gene to a GAL4 protein and tested its repressive activity in the T-REx system^40^ in which HEK293 T-REx cells contain a stably integrated luciferase (Luc) reporter gene under the control of the thymidine kinase (TK) basal promoter with five GAL4 UAS (Supplementary Fig. 15). Tetracycline-induction led to a significant decrease in luciferase expression linked to TaSETup1, which was lost when the mutant H206F was used (Fig. 7c and Supplementary Fig. 15). Additional experiments using flow cytometry sorting (FACS) of low and high H3K18me1 populations during merogony supported our conclusions that parasite differentiation is accompanied by dynamic changes in histone methylation and changes in TaSETup1 expression (Supplementary Fig. 16).

## Discussion

The role of epigenetic mechanisms and histone post-translational modifications in regulating gene transcription and cellular differentiation is well-established for a broad range of mammalian genomes and model organisms^21,22^. Indeed, the enzymes responsible for methylating or acetylating lysine residues in histone tails are identified as targets for drug therapies in a wide range of human pathologies including cancer. However, relatively little is known about how parasite genomes are regulated and the mechanisms controlling differentiation. The paucity of transcription factors in the apicomplexan genomes, suggests that epigenetic mechanisms could play key regulatory roles^25,26^.

This study presents the first insights into how dynamic changes in epigenetic marks might regulate gene expression and stage differentiation in *Theileria* parasites. We report the first characterization of mono-methylation of the H3K18 residue in parasite histone tails. Our experimental evidence includes immunofluorescence staining of individual cells and immunoblot analysis with three independent and specific antibodies, reinforced by flow cytometry experiments and mass spectrometry analysis with a candidate recombinant methyltransferase enzyme. The H3K18me1 was observed in B cells and macrophages infected with either *T. annulata* or *T. parva* parasites and decreased upon treatment with the thericidal Buparvaquone drug. These results suggest that H3K18 methylation is a general marker of *Theileria* schizonts. Future experiments with primary clinical samples from infected cows will be interesting to test when the H3K18me1 appears during the parasite life cycle. Our experiments with parasite differentiation towards merozoites suggest that H3K18me1 may prevent merogony and advancement through the parasite life cycle. Our results are consistent with a model in which the repressive function of H3K18me1 is lost on some loci during the differentiation to merozoites and that this allows the upregulation of a subclass of merogony-associated genes. Notably, the 87 proteins in the M2M_up:Cluster IV group include several potential secreted proteins (including Tpr and SfiI gene families) as well as three candidates resembling rhoptry proteins (TA05870, TA16660, and TA19445) which are good candidates for proteins involved in the invasion. We characterized the *M2M13* gene encoding a potential rhoptry neck protein as a test case. The observation that expression of these *M2M* genes and merogony are affected by drug treatments (KDMi inhibitors) suggests that these drugs may be potential anti-parasite reagents that merit testing using in vivo models of infection.

Currently, relatively little is known about gene regulation in *Theileria* genomes. There is a paucity of transcription factors in all apicomplexan genomes. It is possible that some of the *Theileria* ApiAP2 genes could be stage-transition determinants^19^, and it is noteworthy that some are also in the list of cluster IV genes (Supplementary Table 3). This is interesting as ApiAP2 changes have been linked to *Theileria* differentiation and the PfAP2-G homologue drives an epigenetically regulated master switch that initiates gametocytogenesis in *Plasmodium*^19,41,42^. The link between epigenetic and transcription factor networks in regulating parasite differentiation merits further investigation.

In addition to the novel H3K18me1 epigenetic mark, we identified TaSETup1 as the first SET-domain enzyme capable of H3K18 mono-methylation and the first characterized epigenetic actor in the *Theileria* genome. As there are only five SET-domain proteins in *Theileria* genomes, it is likely that parasite methyltransferases are multifunctional, with diverse substrate specificities. Furthermore, there is an emerging interest in non-histone targets^43^ and TaSETup1 homologues in other apicomplexa species that may have non-histone substrates. Interestingly, the *Toxoplasma* homologue TgAKMT is a cytoplasmic protein important for parasite motility^44,45^ and the *Plasmodium* homologue PfSET7 can methylate H3K4 and H3K9, but also has cytoplasmic functions^46^. Nonetheless, H3K18 methylation has been detected in these parasites^31^ and these enzymes could have both cytoplasmic and nuclear functions depending on the parasite life stage. Indeed, TaSETup1 resembles SMYD proteins that have both cytoplasmic and nuclear targets and functions^34^. Studies of parasite epigenetics shed new light on gene regulation mechanisms; another example is the recent discovery of mono-methylated H4K31^47^ in *Toxoplasma* parasites. Methyltransferases represent versatile regulatory enzymes and the drugs^23^ being developed to block methylation in cancers could be repurposed for infectious diseases. The study of epigenetic regulation in *Theileria* is likely to be rich and fruitful^48^ and our results already suggest that targeting the methylation machinery to block merogony could be a promising therapeutic strategy.

## Methods

### Cell culture and treatment

All bovine cell lines were previously documented: TBL3 cells were derived from in vitro infection of the spontaneous bovine B lymphosarcoma cell line, BL3, with Hissar stock of *T. annulata*. TaC12^27^ is a line of *T. annulata*-infected bovine macrophages. The TpMD409 lymphocyte cell line is infected with *T. parva*. Cells were cultured in RPMI 1640 (Gibco-BRL), supplemented with 10% heat-inactivated foetal bovine serum (FBS), 4 mM l-glutamine, 25 mM HEPES, 10 mM β-mercaptoethanol and 100 mg/ml of penicillin/streptomycin in a humidified 5% CO_2_ atmosphere at 37 °C. HEK 293T-RexLuc cells were grown in DMEM supplemented with 10% FBS and 100 mg/ml of penicillin/streptomycin in a humidified 5% CO_2_ atmosphere at 37 °C. The anti-parasite drug Buparvaquone (BW720c) was used at 50 ng/ml for 48 h (Chemos GmbH, ref: 88426-33-9). The KDM inhibitor (KDMi) is the well-characterized, broad-spectrum lysine demethylase inhibitor tranylcypromine (TCP) (a generous gift from A. Mai, Sapienza University, Rome) used at 1 µM, 48H and the KDAC inhibitor (KDACi) 24 h at 40 nM (compound FR235222, a generous gift from MA. Hakimi). The KDM inhibitor (KDMi) is used at 1 µM, 48 h and the KDAC inhibitor (KDACi) 24 h at 40 nM (compound fr235222 from MA. Hakimi, IAB, Grenoble, France).

### Merogony induction

Macroschizont-infected TaC12 cells were induced to differentiate to merogony by increasing the culture temperature to 41 °C^32^. Cells were passaged each time they reached confluence and 2 × 10^6^ cells collected at day 0 (macroschizont stage) and day 8 (merogony stage) for RNA extraction and 4 × 10^3^ cells per immunofluorescence at the same time-points.

### Immunofluorescence analysis

Cultured *T. annulata*-infected macrophages (TaC12) or B cells (TBL3) were washed with PBS containing 1 mM EDTA and 3 × 10^4^ cells per slide were centrifuged with Cytospin (10 min at 277×*g*) to adhere to the slide. Cells were fixed in 3.7% paraformaldehyde for 15 min and subsequently permeabilised in 0.2% Triton X-100 (prepared in PBS) for 10 min. Fixation, permeabilisation and all the following steps were carried out at room-temperature. Slides were blocked with PBS 0.2% Tween (PBST)–1% BSA for 30 min. Primary antibodies were diluted in PBST and incubated for 1 h at the following dilutions: rabbit anti-H3K18me1 (ab177253, Abcam), 1:5000; rabbit anti-H3K18ac (9675S, Cell signaling) 1:800, rabbit anti-H3K4me3 (pAb-003-050, Diagenode) 1:200. Cells were subsequently washed three times with PBST and incubated with secondary antibody for 30 min at the following dilutions: Alexa594-conjugated donkey anti-rabbit antibody, 1:1000. Cells were washed three times with PBST and finally, mounted on coverslips adding ProLong Diamond Antifade Mountant with DAPI mounting reagent (Thermo Fischer Scientific). Samples were analysed using a Leica DMI 6000 epifluorescence microscope. Images were generated and processed using Metamorph and ImageJ software. For H3K18ac and H3K18me1 intensity quantification in the parasite, for each cell, we calculated the mean intensity in the entire parasite using ICY software, divided by the number of parasite nuclei per host cell. The counting of cells in the macroschizont or merogony stage was done using ImageJ. We defined a threshold for the Schizont/Merogony cycle stage, at 80 parasites per cell.

### Protein extraction and Western blot analysis

For all the cell lines, 2 × 10^7^ cells were collected and histone extraction was performed using the Abcam Kit protocol. Histones were resolved on NuPage 4–12% acrylamide gradient SDS–PAGE gel (Invitrogen) and transferred onto nitrocellulose membranes in Tris–glycine transfer buffer. Membranes were blocked and incubated overnight at 4 °C with the primary antibodies: H3K18me1(ab177253) 1:20,000; H3K18ac (ab1191) 1:2000; H3 (ab1791) 1:10,000. Membranes were incubated with the appropriate secondary antibody coupled to horseradish peroxidase (HRP), revealed using West Dura kit and the Licor detection system.

### Luciferase reporter activity

HEK 293T-RexLuc cells were grown in DMEM supplemented with FBS Tetracycline-free (EuroBio) and transfected with the pcDNA4-TO-Gal4-G9a, pcDNA4-TO-Gal4-TaSetup1, pcDNA4-TO-Gal4-PrSet7, and pcDNA4-TO-Gal4-TaSETup1-H207F plasmids using lipofectamine 2000. The proteins were induced 5 h post-transfection with Tetracycline (final concentration of 1 µg/ml). Transfection efficiencies were normalized to Renilla activity by co-transfection of a pRL-TK Renilla reporter plasmid at 150 ng. Luciferase assays were performed 36 h post-induction using the Dual-Glo Luciferase assay system (Promega) in a microplate luminometer. The percentage of luciferase activity was represented as the ratio Firefly/Renilla luminescence, compared with the non-induced transfected cells.

### Plasmids and transfection

pCDNA4-TO-Gal4-G9a and pcDNA4-TO-Gal4-PrSet7 were a gift from S. Ait-Si-Ali. pcDNA4-TO-Gal4TaSETup1 and pcDNA4-TO-Gal4-TaSETup1-H207F were generated from pcDNA4-TO-Gal4-G9a cloning TaSETup1 cDNA from *Theileria* infected macrophages RNA, with restriction enzymes *EcoR*I and *Not*I. Each plasmid was transfected in HEK 293T-RexLuc cells with Lipofectamine 2000 in a concentration gradient (0.5, 1 and 2 µg). pRL-TK Renilla reporter plasmid was co-transfected at 150 ng.

### RNA extraction and RT-qPCR

For all cell conditions, total RNA was extracted using a Nucleospin RNA extraction kit (MachereyNagel) following the manufacturer’s protocol. 1 µg of total RNA was reverse transcribed with Superscript III Reverse transciptase Kit (Invitrogen). Real-time quantitative PCR was performed to analyse relative gene expression levels using SyberGreen Master Mix (Applied Biosystem) following the manufacturer’s protocol. Relative expression values were normalized with housekeeping gene mRNA *HSP70*. Primer sequences are listed in Supplementary Table 5.

### RNA-Seq analysis

5 × 10^6^ cells (BL3 or TBL3) were used as starting material to extract RNA. Extraction was performed following the TRI-reagent (SIGMA, T-9424) protocol. Library preparation and sequencing were performed at the GenomIC’ sequencing facility (https://www.institutcochin.fr/core_facilities/genomesequencing-studies?set_language=en). Briefly, poly-A Library preparation was done using the Illimuna TrueSeq stranded protocol and paired end 75 bp sequencing was performed on a Illumina NextSeq 500 to a depth of over 40M reads per sample. Read-quality control was performed using fastQC (v0.11.7) and Rsubread^49^ (v1.26.1) qualityScore function. Read mapping was performed using the Rsubread align function on an indexed version of *T. annulata* genome assembly ASM322v1.32. RPKM values were obtained using the featureCounts function from the Rsubread package using the same ASM322v1.32 annotations. Visualisation and snapshots of bam files were performed using IGV (v2.3.91) and further modified using inkscape (v0.92.3). Kruskal–Wallis and pairwise Wilcoxon Statistical testing on RPKM values from cluster I–V was performed using the ggpubr (v0.2) R package.

### Chromatin immunoprecipitation and next-generation sequencing

BL3 and TBL3 cells (2 × 10^7^) were fixed for 10 min with 1% formaldehyde at room temperature. Fixation was stopped with 125 mM glycine for 5 min. Fixed cells were washed 2× with cold PBS. After washes, cells were resuspended in 1 ml cell lysis buffer (Hepes pH 7.8 25 mM, MgCl_2_ 1.5 mM, KCl 10 mM, DTT 1 mM, NP-40 0.1%), incubated on ice for 10 min, centrifuged (492×*g*, 5 min, 4 °C) before resuspension in 1 ml Nuclear Lysis Buffer (Hepes pH 7.9 50 mM, NaCl 140 mM, EDTA 1 mM, Triton X100 1%, Na-deoxycholate 0.1%, SDS 0.5%). After the nuclei were obtained, the chromatin was sheared with a Bioruptor pico to yield 100–400 bp DNA fragments; the sonication conditions were high intensity 30 s ON/30 s OFF, for 12 min. Following centrifugation (10 min, 24,104×*g*) the supernatant was used for immunoprecipitation. Sheared chromatin were incubated overnight at 4 °C with anti-H3K18me1 (Abcam 177253), anti-H3K18Ac (Cell Signaling 9675S), anti-H3K4me3 (Millipore 07-473), anti-H3K36me3 (ab9050), or IgG isotype control (Cell Signaling 27295). The immunoprecipitation was carried out using Dynabeads protein G (Thermo Fisher Scientific) for 3 h at 4 °C. Immunoprecipitates were washed four times with IP buffer, once with wash buffer (Tris pH 8 20 mM, LiCl 250 mM, EDTA 1 mM, NP-40 0.5%, Na-deoxycholate 0.5%), and twice with elution buffer (Tris pH 8 20 mM, EDTA 1 mM). Ten immunoprecipitated chromatin was eluted by incubating beads with elution buffer supplemented with 1% SDS at 65 °C. Both input and ChIP DNA were then treated with RNase A for 1 h at 37 °C, followed by the addition of Proteinase K and overnight incubation at 65 °C to reverse cross-link. DNA was then purified with NucleoSpin Gel and PCR clean-up kit (MachereyNagel) following the manufacturer’s instructions.

ChIP-Seq libraries were prepared using the MicroPlex v2 kit (Diagenode) according to the manufacturer’s instructions. 10 µl of DNA material was used and a 10-cycle PCR was performed as a final amplification of the libraries. Libraries were sequenced on a NextSeq 500 system (Illumina). A 75-base single-end run was performed, with the libraries loaded as a 2pM equimolar pool with 1% of internal control sequences (PhiX–Illumina). 594 million reads were generated with Q30 = 86.37%. Raw reads were converted to Fastq files and their quality assessed using Aozan (version 2.2.1)39. Read-quality control was performed using fastQC (v0.11.7) and Rsubread^49^ (v1.26.1) quality score function. Read mapping was performed using the Rsubread align function on an indexed version of Theileria annulata genome assembly ASM322v1.32. Biological replicates correlation was assessed using PCA and Spearman/Pearson correlation coefficient computation using deepTools^50^ (v3.1.1) bamCoverage, multiBamSummary and plotCorrelation tools. K-means clustering on H3K18me1 ChIPseq analysis was performed using the computeMatrix and plotHeatmap tools. Circos plot of ChIP-seq and RNA-seq experiment was done using circlize^51^ (v0.4.5).

### Gene set overlaps

Representation factor and associated probability between genes from Cluster IV and stage-specific differentially expressed genes (from reported study^19^) were calculated using the software available at http://nemates.org/MA/progs/overlap_stats.html. Briefly, the representation factor corresponds to the number of overlapping genes divided by the expected number of overlapping genes drawn from two independent groups. Associated probability was computed using an exact hypergeometric test. Details of the computations can be found at http://nemates.org/MA/progs/representation.stats.html. R and ggplot2 packages were used to produce several figure panels.

### Phylogenetic analysis

SET domain proteins were retrieved from interproDBv66^52^ using the SET domain identifier IPR0014. *Homo sapiens* SET proteins were further curated to include only some representatives of the major SET families. Proteins were aligned using mafft^53^ v7.245 with option --localpair --maxiterate 1000 --ep 0. Alignment was examined using Jalview^54^ v2.10.5 and phylogenetic tree inferred with Ultra-Fast bootstrap and alrt branch support using iqtree^55–57^ v1.5.5 with options –m TEST -bb 1000 –alrt 1000. FigTree v1.4.3 was used for tree annotation. Computations were performed using Docker containers available at https://hub.docker.com/u/parisepigenetics/.

### Reannotation of parasite genes

*T. annulata* protein sequences were submitted to the blast2GO^58^ pipeline for annotation using standard parameters.

### Cloning and site-directed mutagenesis

Total cellular RNA from infected macrophages (TaC12) was converted to cDNA using Phusion high fidelity DNA polymerase (ThermoFisher). The open reading frame of TaSETup1 (TA06820, piroplasmaDB) (refseq_948938) was PCR amplified with Q5 high fidelity DNA polymerase (NEB) and cloned by BP recombination reaction into entry clone pDONR207 using GATEWAY^®^ cloning technology. The positive entry clone containing TaSETup1 was shuttled into pDEST17 His Tag Expression vector by LR recombination reaction using GATEWAY^®^ cloning technology. For site-directed mutagenesis of TaSETup1, Histidine 206 residue was mutated into phenylalanine using Phusion Site-Directed Mutagenesis Kit (Thermofisher). The resulting positive clone was transformed to competent *E. coli* host HI-control BL21 (DE3) cells for protein expression and purification.

### Protein expression and purification

A single colony of pDEST17 TaSETup1 WT, or H206F, was inoculated into 5 ml of LB broth containing 100 µg/ml ampicillin. The overnight culture was transferred to 1 l of fresh medium and was grown at 37 °C until OD value of 0.7 at 600 nm was reached. Isopropyl β-d-1-thiogalactopyranoside (IPTG) was added to a final concentration of 750 µM and grown overnight at 16 °C. Cells were harvested by centrifugation at 2490×*g* for 20 min and the pellet re-suspended for 30 min at 4 °C under agitation in cold lysis buffer (300 mM NaCl in PBS 1×, pH 8, triton 1%, lysozyme 1 mg/ml, 10 mM imidazole, cOmplete^TM^ Protease Inhibitor Cocktail). The cells were disrupted by sonication on ice and clarified by centrifugation at 16,000×*g* for 30 min at 4 °C. Proteins carrying the histidine tag were purified using HIS-Select^®^ Nickel Affinity Gel. Briefly, the clarified lysate was incubated with affinity beads for 3 h at 4 °C under agitation and then transferred to a chromatography column. After extensive washing, the histidine-containing protein was eluted from the column using 5 column volumes of elution buffer (300 mM NaCl in PBS 1×, pH 8, 300 mM Imidazole). Samples were subjected to buffer exchange into the low salt buffer (Tris–HCl 50 mM, NaCl 50 mM, pH 8) using PD-10 Desalting Columns containing Sephadex G25 resin (GE Healthcare). Desalted samples were concentrated using an Amicon Ultra centrifugal filter units (cutoff 10 kDa, EMD Millipore) and subjected to gel filtration using a HiLoad 16/60 Superdex 200 size-exclusion column using an AKTA purifier system (Cytvia). The sample was eluted using the low salt buffer and fractions containing HIS-tagged protein were pooled, concentrated using *Amicon* Ultra centrifugal filter units (cutoff 10 kDa) and analysed by SDS–PAGE gel electrophoresis and Coomassie staining.

### RP-UFLC-based separation and quantification of the fluorescein-labelled peptide substrate of TaSETup1 (FAM-H3K18) and its methylated products (FAM-H3K18me)

A 9-amino-acid peptide derived from the sequence of human histone H3.1 protein and containing the lysine 18 residue was synthesized and conjugated to fluorescein amide (FAM) on its N-terminus and modified by amidation (NH2) on its C-terminus (Proteogenix, France). The lysine 14 was mutated to arginine in order to only monitor lys-18 TaSETup1-dependent methylation. The peptide was as follows: FAM- RAPRK_18_QLAT-NH2. A mono, di, and tri-methylated different forms of H3K18 (H3K18_me1/me2/me3_) peptide was also synthesized and used as standard. The lysine methyltransferase reaction was carried out overnight at room temperature in methylation buffer (Tris 50 mM pH 8, 50 mM NaCl, 1 mM DTT) containing 75 µM of the peptide substrate, 3 µM of enzyme and with or without 200 µM of S-Adenosyl methionine (SAM). Samples containing H3K18 peptide (substrate) and its methylated forms (products) were separated by RP-UFLC (Shimadzu) using Kromasil 100-5-C18 column 4.6 × 250 mm, 5 µm particle size at 40 °C. The mobile phase used for the separation consisted of two solvents. Solvent A containing water with 0.1% perchloric acid (HClO_4_) and solvent B containing acetonitrile with 0.12% trifluoacetic acid (TFA). Separation was performed by an isocratic flow as followed: 80% A/20% B, rate of 1 ml/min, time of run = 27 min. H3K18 peptide (substrate) and its methylated forms (products) were monitored by the fluorescence emission (*λ* = 530 nm) after excitation at *λ* = 485 nm and quantified by integration of the peak absorbance area, employing a calibration curve established with various known concentrations of peptides.

### Histone methyltransferase in vitro assays

Recombinant histone H3 (NEB), H3 from calf Thymus (Sigma), or core histone purified from chicken erythrocytes (Sigma) (500 ng) were mixed with or without S-adenosyl methionine (100 µM final) in 1× HMT buffer containing 50 mM Tris pH 8.0, 20 mM KCl, 5 mM MgCl2, 5% glycerol, 1 mM DTT in a final volume of 25 μl and incubated at room temperature for 2 h. Samples mixtures were immobilized on nitrocellulose membrane using Bio-Dot^®^microfiltration apparatus (Bio-Rad) and blocked with 5% non-fat milk in TBS-Tween for 1 h. After three washes, membranes were incubated with antibodies against H3 (1:2000), H3K18me1 (1:1000), at 4 °C overnight. Membranes were washed three times for 10 min and incubated with a 1:5000 dilution of horseradish peroxidase-conjugated anti-rabbit antibody for 1 h at room temperature and developed with the Pierce ECL Western Blotting Substrate according to the manufacturer’s protocol.

### Sample preparation for LC–MS/MS analysis

50 µg of an H3-derived 14-mer peptide flanking the lysine 18 (GGKAPRKQLATKAA-NH2, Proteogenix) were incubated with 3 µg TaSETup1 and with or without 1 mM SAM for 2 h at room temperature. The reaction was then stopped by cooling and the samples were analysed by LC–MS/MS as follows.

### LC–MS/MS acquisition

The peptide solution was desalted using ZipTip µ-C18 Pipette Tips (Millipore) and analysed by an Orbitrap Tribrid Fusion mass spectrometer in positive mode (Thermo Scientific) coupled to a Nano-LC Proxeon 1200 equipped with a NSI EASY-spray ion source (Thermo Scientific). Peptides were separated by liquid chromatography with the following parameters: Acclaim PepMap100 C18 pre-column reversed-phase (2 cm, 3 μm, 100 Å), EASY-spray C18 column reversed phase (P/N ES805A, 75 cm, 75 μm, 2 μm, 100 Å), 300 nl/min flow rate, gradient from 95% solvent A (water, 0.1% formic acid) to 40% solvent B (80% acetonitrile, 0.1% formic acid) over a period of 120 min, followed by a column regeneration of 20 min, giving a total run time of 140 min. Peptides were analysed in the Orbitrap cell, in full ion scan mode, at a resolution of 120,000 with a mass range of *m*/*z* 350–1550 and an AGC target of 4 × 105. Fragments were obtained by high collision-induced dissociation (HCD) activation with a collisional energy of 27%, and a quadrupole isolation window of 1.6 Da. MS/MS data were acquired in the Ion trap in a Top-Speed mode with 3 s cycles, with an AGC target of 1 × 104 and with a dynamic exclusion of 60 s. MS/MS of most intense precursor were firstly acquired. Peptides with charge states = 1–8 and unassigned charge states were included for the acquisition. The maximum ion accumulation times were set to 100 ms for MS acquisition and 35 ms for MS/MS acquisition.

### LC–MS/MS data processing

The LC–MS/MS.raw files were processed using the Sequest search engine of Proteome Discoverer 2.4 (Thermo Fisher Scientific). The peptide identification was done in No-enzyme mode with a custom database containing only the peptide sequence. The precursor mass tolerance was set to 7 ppm and the fragment mass tolerance to 0.5 Da. Validation of spectra was done with the “Fixed value PSMs validator” node, which performs validation of PSMs (Peptide Spectrum Matches) based on score thresholds defined for the search nodes.

On proteome Discoverer 2.4, the following dynamic modifications were searched: Methylation (K), Dimethylation (K), Trimethylation (K), amidated (C-terminus of peptide) and HCysThiolactone (K).

### Characterization of H3K18me1 antibody specificity

2 µg of 5-FAM coupled short peptides (Proteogenix) flanking the unmodified, monomethylated, dimethylated, trimethylated or acetylated lysine of interest (H3K4: ARTKQTARRSK, H3K9: RQTARKSTGG, H3K14: STGGKAPRR, H3K18: RAPRKQLAT, H3K27: TKAARKSAPAT and H3K36:

TGGVKRPHR) were transfered on nitrocellulose membrane using Bio-Dot^®^microfiltration apparatus (Bio-Rad) and blocked with 5% non-fat milk in PBS-Tween for 1 h. Membranes were then incubated with antibodies against H3K18me1 (Abcam #ab177253, Active Motif #31259 and a home-made antibody provided by Jane Mellor’s laboratory) (1:10,000) or H3K36me3 (Abcam #ab9050) (1:10,000) at 4 °C overnight. Membranes were washed three times with PBS-Tween for 10 min and incubated with a 1:20,000 dilution of horseradish peroxidase-conjugated anti-rabbit antibody for 1 h at room temperature. Membranes were finally developed with the Pierce ECL Western Blotting Substrate according to the manufacturer’s protocol.

### Flow cytometry analysis

Cells were washed once with PBS and then fix overnight in 0.5% PFA in PBS at 4 °C. After centrifugation (5 min at 300×*g*, 4 °C), cells were washed in PBS, recentrifuged and resuspended in 0.1% Triton-X (5 min) then washed once in FACS Buffer (cold PBS, 20 mM Hepes and 0.5% BSA) Then, primary antibodies were added in FACS buffer and incubated 20 min at 4 °C. Cells were washed with FACS buffer and repeat with secondary antibody (at the same dilution conditions as immunofluorescence experiments above). Cells were washed again with FACS buffer and resuspended in 200 µl of FACS buffer for flow cytometry analysis.

The sequences of all primers and oligonucleotides used in this study are listed in Supplementary Table 5.

### Reporting summary

Further information on research design is available in the Nature Research Reporting Summary linked to this article.

## Supplementary information

Supplementary Information

Reporting Summary

## Data Availability

All data generated or analysed during this study are included in this published article. The RNA-Sequencing and ChIP-Seq data have been deposited to the ENA database with the study identifier #PRJEB33792. The Zenodo access link is provided (10.5281/zenodo.3370034) DOI number: 10.5281/zenodo.3370034. Content description: This repository provides bigwig files for ChIP and RNA sequencing experiments in *Theileria annulata*. DeepTools generated Coverage files, ReadCount normalised files and SES normalised files are provided. Also provided are genome and annotation files for visualisation with IGV software. The Mass Spectrometry proteomics data were deposited in the ProteomeXchange Consortium via the PRIDE partner repository with the dataset identifier PXD024599. Source data are provided with this paper.

## Source data

Source Data
