# Supplementary material for: Dynamic methylation of histone H3K18 in differentiating Theileria parasites
Source: Nat Commun. 2021 May 28;12:3221. doi: 10.1038/s41467-021-23477-2 (PMC8163883; doi:10.1038/s41467-021-23477-2)
Supplement: Supplementary file 1 — Supplementary Information [file 41467_2021_23477_MOESM1_ESM.pdf]

SUPPLEMENTARY INFORMATION

TITLE

Dynamic methylation of histone H3K18 in differentiating *Theileria* parasites

Authors

Kevin Cheeseman*[1], Guillaume Jannot*[1], Nelly Lourenço*[1], Marie Villares*[1], Jérémy Berthelet[1], Teresa Calegari-Silva[1], Juliette Hamroune[3], Franck Letourneur[3], Fernando Rodrigues-Lima[2], Jonathan B. Weitzman[1]

Supplementary Data Figures 1-17

Supplementary Tables 1-5

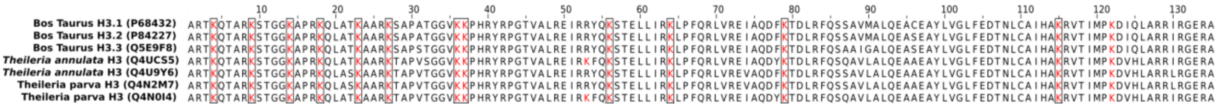

Supplementary Figure 1: Conserved histone sequences in cows and *Theileria* parasites. Multiple alignments of the protein sequences of N-terminal tails of *Bos taurus* Histone H3.1, H3.2 and H3.3, together with the two histone H3 proteins in the *T. annulata and T. parva* genomes. Highly conserved lysine residues (K) are highlighted in red and the UniProt accession numbers indicated.

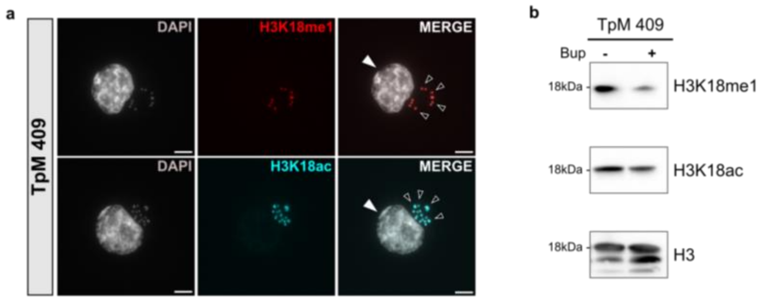

Supplementary Figure 2: Methylation of H3K18 in *T.parva* parasites.

a. Immunofluorescence analysis of H3K18me1 (red) and H3K18ac (cyan) staining of TpM409 cells infected with *T. parva*. Host and parasite nuclei are stained with DAPI (grey). The solid white arrowheads indicate the host cell nucleus and empty white arrowheads point to the parasite nuclei. Leica microscope, 100X, Scale bar = 5µm.

b. Western blot analysis of TpM409 cells treated with Buparvaquone (Bup), a theilericidal drug. H3 was used as a loading control.

All these experiments were performed three times independently with similar results; these data show one of these three times. Full scans of blots and immunolfuorescence replicates are included in the Source Data file.

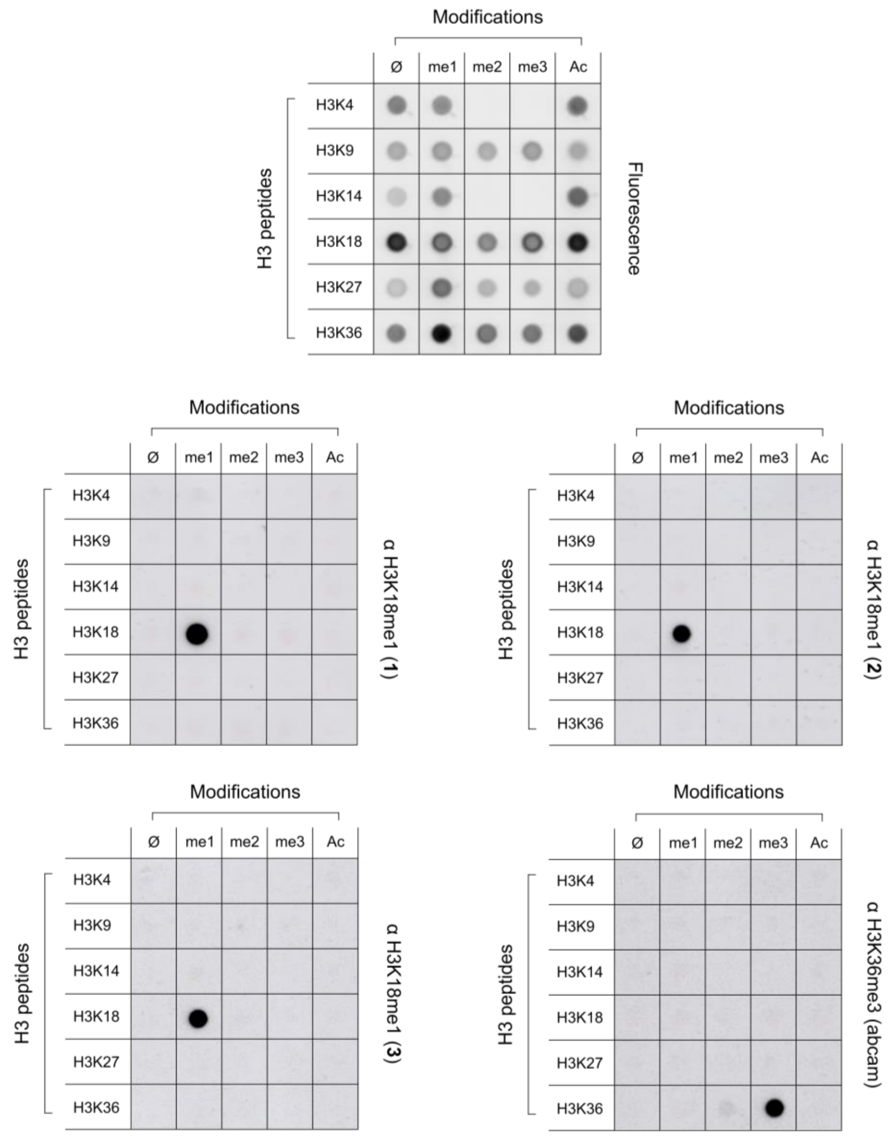

Supplementary Figure 3: Characterization of antibody specificity using H3-derived modified peptides.

Results of dot-blot analysis testing three independent antibodies recognizing mono-methylated H3K18 against a range of peptide sequences. 2 µg of 5-FAM coupled short peptides flanking the unmodified, methylated or acetylated lysine of interest (namely H3K4: ARTKQTARRSK, H3K9: RQTARKSTGG, H3K14: STGGKAPRR, H3K18: RAPRKQLAT, H3K27: TKAARKSAPAT and H3K36: TGGVKRPHR) were transferred onto a nitrocellulose membrane and immunodetected using either antibodies against H3K18me1 (1: Abcam #ab177253, 2: Active Motif #31259, 3: a home-made antibody provided by Jane Mellor's laboratory) or H3K36me3 (Abcam #ab9050). Fluorescence of peptides was also detected as a dotting control.

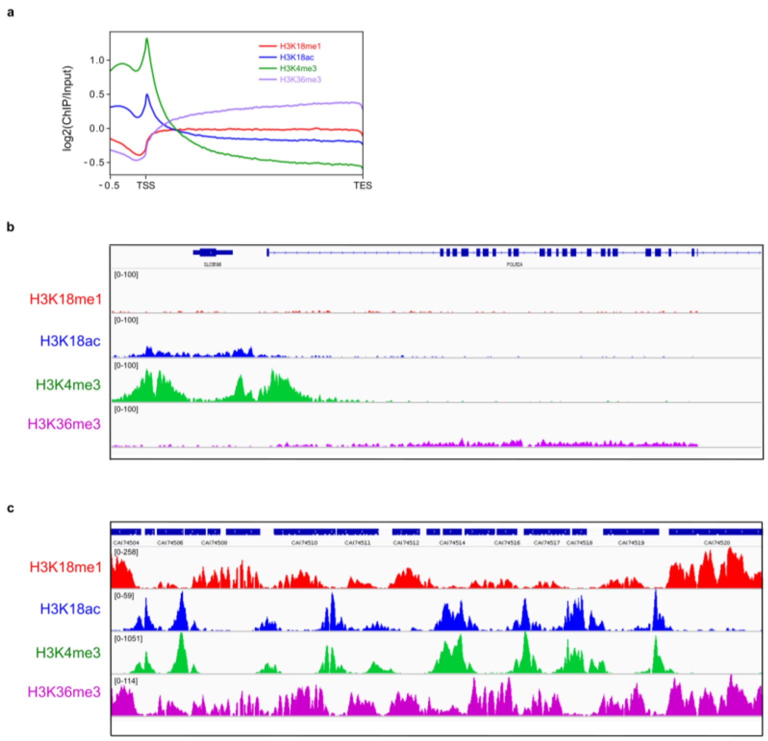

Supplementary Figure 4: H3K18me is not found on gene bodies on the bovine genome.

a. Average occupancy profiles for H3K4me3 (green), H3K18ac (blue), H3K18me1 (red) and H3K36me3 (purple) around the transcriptional start site (TSS) of all *Bos taurus* genes. X-axis: genome coordinates starting from 500 bp before the TSS to the TES. Y-Axis: log2 (ChIP/Input). H3K4me3 and H3K18ac profiles display enrichment around the TSS region, whereas H3K36me3 is depleted at the TSS and shows an enrichment over the gene bodies. H3K18me1 is not enriched on the bovine genome.

b. Chromatin ChIP-Seq profiles over a 25 kb-long representative region of the *Bos taurus* genome. Top track: Annotations, H3K18me1 (red), H3K18ac (blue), H3K4me3 (green), and H3K36me3 (purple).

c. Chromatin ChIP-Seq profiles over a 25 kb-long representative region of the *T. annulata* genome. Annotations, H3K18me1 (red), H3K18ac (blue), H3K4me3 (green), and H3K36me3 (purple).

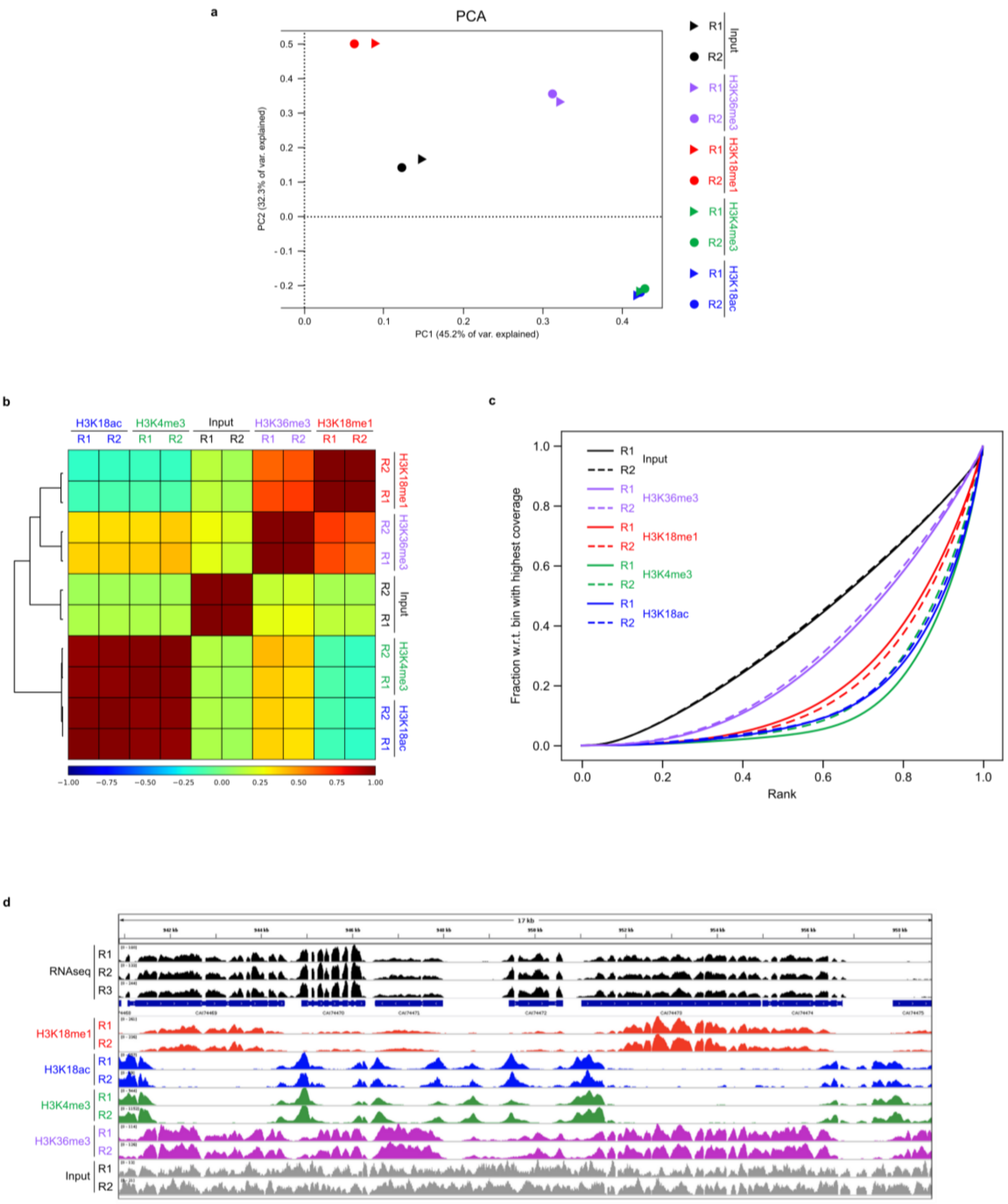

Supplementary Figure 5: Spearman correlation plot of ChIP-Seq data on the parasite genome.

a. Graph of the Principal component analysis (PCA) for the various ChIP duplicate samples demonstrating good correlation between replicates.

b. Clustered Heatmap of H3K18me1, H3K18ac and H3K4me3 and H3K36me3 Spearman correlation coefficients for read mapping from the ChIP-Seq analysis of the parasite genome. Clustering was performed using the nearest point algorithm.

c. Results of the deepTools plotFingerprint analysis to evaluate ChIP enrichment.

d. Genome browser tracks depicting epigenetic profiles in replicate samples (these data are merged in the presentation in Figure 2e). RNAseq (three replicas, black), H3K18me1 (red), H3K18ac (blue), H3K4me3 (green), H3K36me3 (purple) and Input (grey).

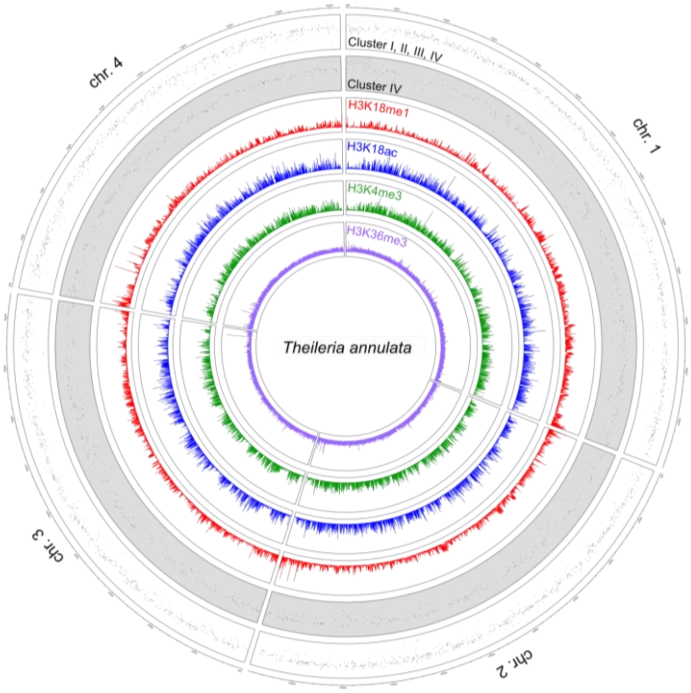

Supplementary Figure 6: Circular chromosomal representation of ChIP-seq and gene expression. Representation of the four chromosomes (chr. 1-4) of the *T. annulata* genome: each circular track showing (from the innermost track): H3K36me3 (purple), H3K4me3 (green), H3K18ac (blue) and H3K18me1 (red) ChIPseq data, the last two tracks show log10(RPKM) gene expression values for genes belonging to cluster IV (light grey background) or to clusters I, II, III & V.

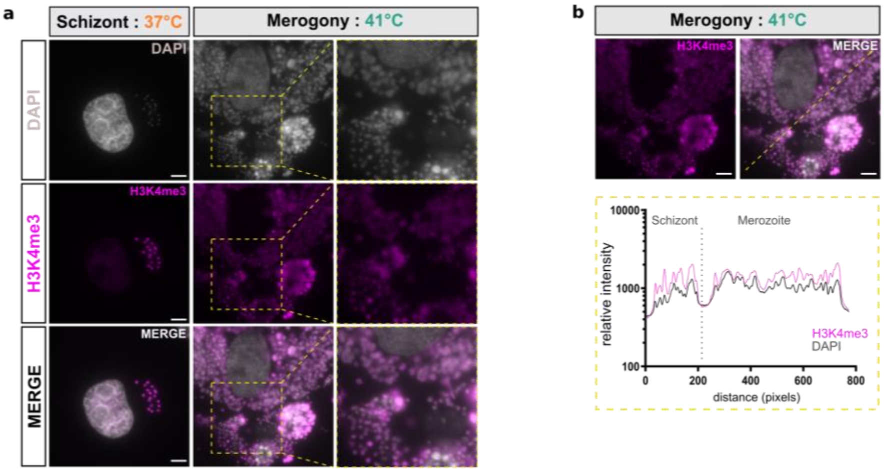

Supplementary Figure 7: H3K4me3 staining upon merogony.

a. Immunofluorescence analysis of TaC12 infected macrophages cultured at 37°C (left) or following merogony induction for 8 days at 41°C (right). Host and parasite nuclei are stained with DAPI (grey) or a specific antibody against H3K4me3 (magenta). Leica microscope, 100X, Scale bar = 5µm.

b. Quantification of immunofluorescence intensity of H3K4me3 (magenta) compared to DNA (grey) showing constant staining across all parasites. The plot profiles represent the yellow line cross-section.

All these experiments were performed three times independently with similar results; these data show a representative example of these three times.

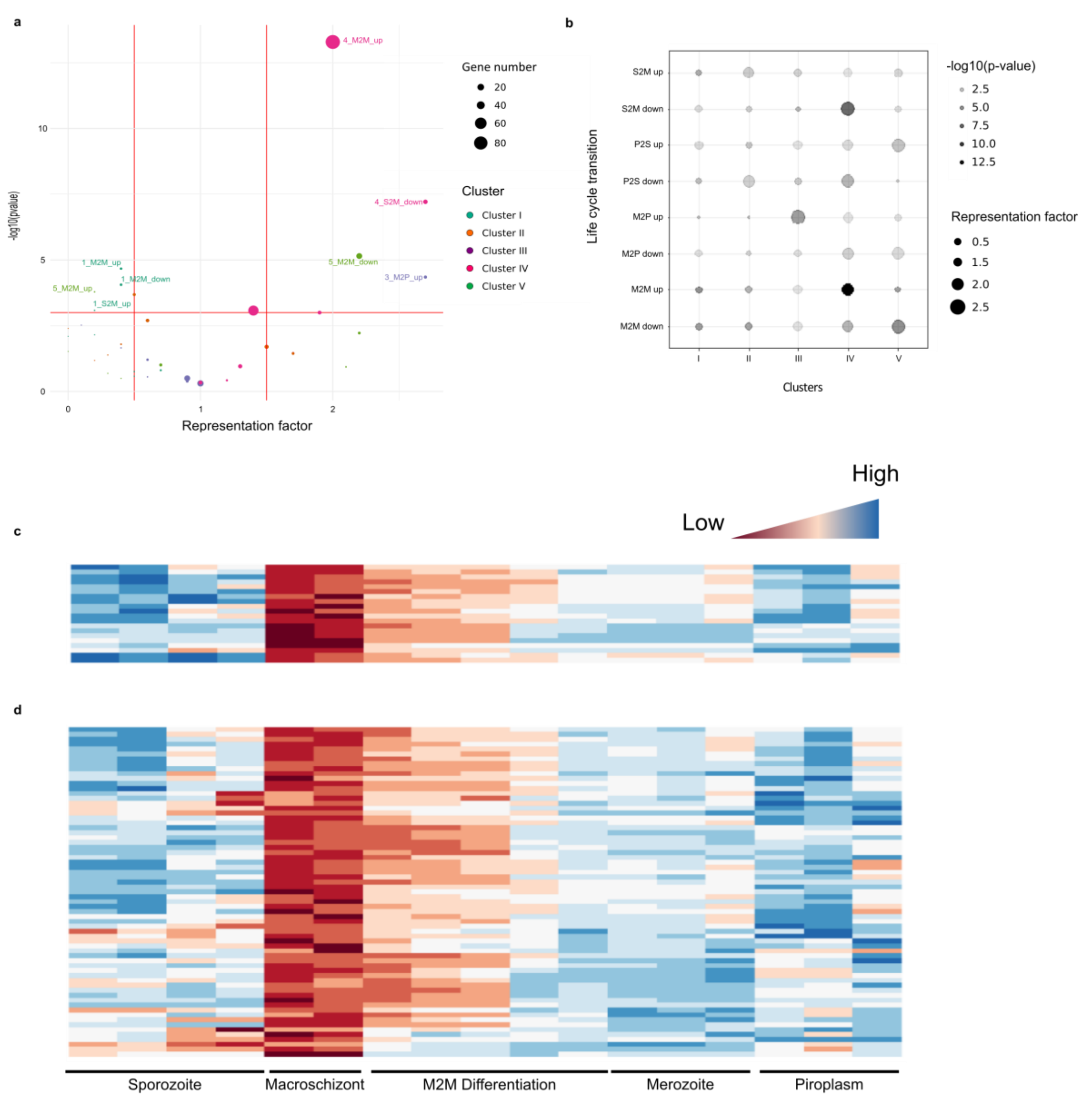

Supplementary Figure 8: Analysis of differentially expressed genes across the parasite life cycle.

a. Chart showing the representation factor as a function of p-value for each life cycle transition as reported by Pieszko *et al*. Dot size is defined by gene number, and colours indicate genes from the different clusters. The vertical red lines indicate representation factors of 0.5 or 1.5 and the horizontal red line indicates a p-value of 0.001. Only groups with a p-value < 0.001 and a representation factor >1.5 or <0.5 are labelled. Labels: M2M_up and M2M_down, macroschizont to merozoite upregulated or down-regulated genes; S2M_up and S2M_down, sporozoite to macroschizont upregulated or down-regulated genes; M2P_up, merozoite to

piroplasm up-regulated genes; and P2S_down, piroplasm to sporozoite down-regulated genes. Notably, the M2M and S2M dots corresponding to Cluster IV are highly significant.

b. Dot chart of representation factors and p-values of overlap between differentially expressed genes in the transition to different life cycle stages derived from a published microarray (Pieszko et al.) and presently derived clusters. Dot transparency is plotted as p-value and dot size displays the representation factor: S2M_up and S2M_down, sporozoite to macroschizont up-regulated or downregulated genes; P2S_up and P2S_down, piroplasm to sporozoite up-regulated or downregulated genes; M2P_up and M2P_down, merozoite to piroplasm up-regulated or down-regulated genes; and M2M_up and M2M_down, macroschizont to merozoite up-regulated or down-regulated genes.

c. Analysis of gene expression profiles in differentiating parasites and correlation with cluster IV. Investigation of the parasite genes belonging to cluster IV over the course of the life-cycle (sporozoite>macroschizont->differentiation to merozoite -> merozoite->piroplasm) using the microarray data from Piezsko *et al.* (2015). Heatmap analysis of the 20 genes that overlap in the S2M-down:M2Mup: cluster IV subgroup (listed in Table 1).

d. Heatmap analysis of the 67 parasite genes from in the M2M-up: cluster IV subset (listed in Table 2). Heatmap scale based on z-score of expression values from series GSE71307 (https://www.ncbi.nlm.nih.gov/geo/query/acc.cgi?acc=GSE71307), and coloured from red to blue (low expression to high expression).

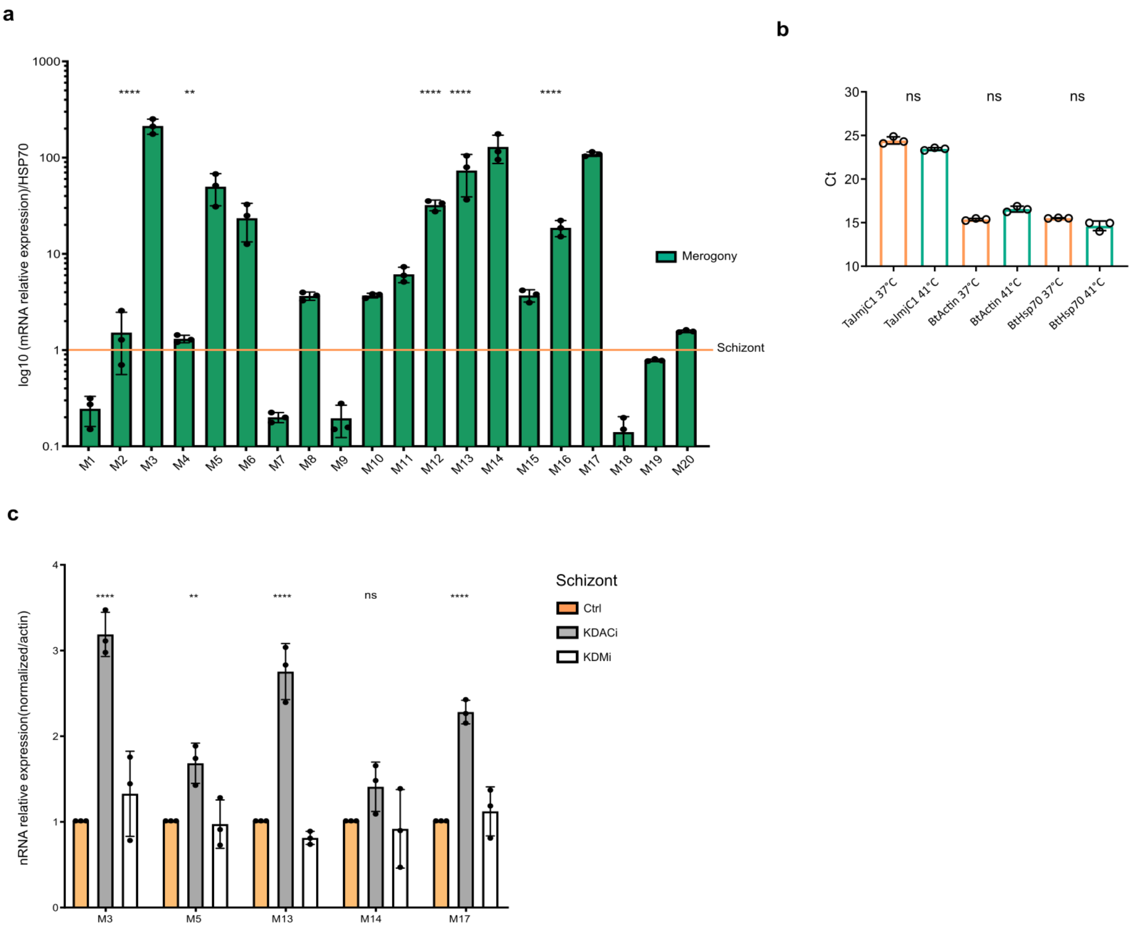

Supplementary Figure 9: Expression of potential stage-associated Cluster IV genes.

a. Expression analysis (RT-qPCR) of the 20 identified *M2M* genes (listed in Supplementary Table 1) in infected TaC12 macrophages, before and after merogony induction culture conditions. The orange line indicates the relative gene expression in cells grown at 37°C (macroschizont stage). The results represent the mean of expression results from three independent experiments. 15 of the *M2M* genes increased in expression upon merogony. Error bars represent the mean values +/- SD. ns p>0.99; * p=0,0249; **** p<0.0001.

b. Expression analysis (RT-qPCR) of control genes (parasite, Ta or bovine, Bt) that do not show changes in expression upon induction of merogony.

c. Expression analysis (RT-qPCR) of selected *M2M* genes following treatment of TaC12 infected macrophages with epigenetic inhibitors KDACi (grey bars) or KDMi (white bars). Results are shown as the mean values +/- SD for three independent experiments with ns p>0.2310; ** p=0,0052; **** p<0.0001.

For all experiments n=3, Statistical Dunnett's multiple comparison test, two-sided: ns=not statistically significant; **** p<0.0001; **p<0.005.

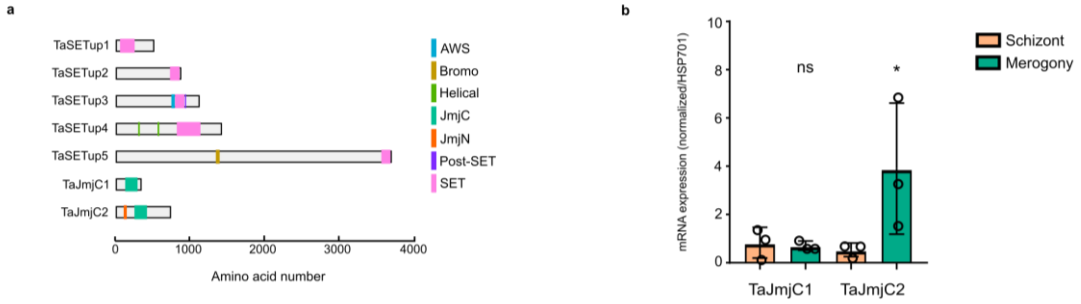

Supplementary Figure 10: Schematic representation of SET domain proteins (TaSETup1-5) and potential demethylases (TaJmjC1-2)

a. Schematic representation of the five putative lysine methyltransferases (listed in Supplementary Table 4) and the two potential demethylases, indicating the presence of the putative catalytic SET domain and other domains, Jumonji domains (JmjC and JmjN), Bromo domain, AWS (Associated With SET) domain, and a post-SET domain.

b. Expression analysis (RT-qPCR) of the two parasite putative demethylases in cells grown at 37°C (schizont stage) or at 41°C (merogony). The results represent the mean values +/- SD for three independent experiments. Statistical Dunnett's multiple comparison test, two-sided: ns=not statistically significant p>0,9876; *p<0.0461

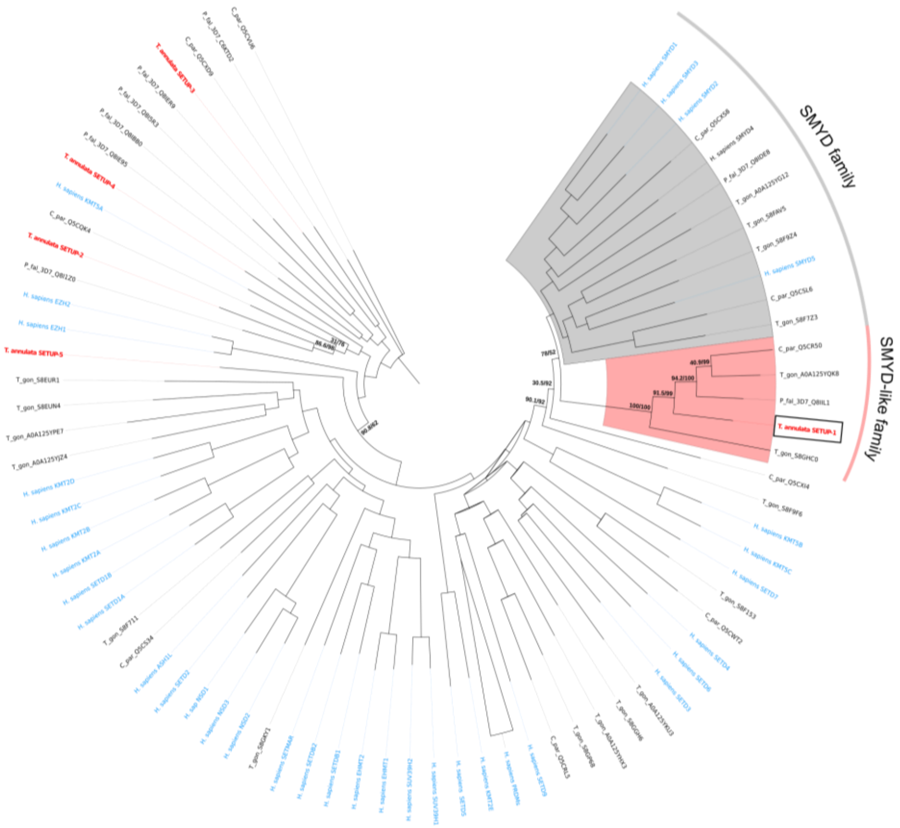

Supplementary Figure 11: Phylogenetic analysis of genes encoding *Theileria* SETup proteins

The tree is constructed with *Homo sapiens* KMT proteins (light blue with their generic names), *T. annulata* TaSETup proteins (red) and other apicomplexan proteins (black) with the species name first followed by their InterPro accession number. P_fal_3D7: *Plasmodium falciparum* 3D7, C_par : *Cryptosporidium parvum*, T_gon *: Toxoplasma gondii*. The SMYD (SET and MYND domain family) and SMYD-like orthologs are shown in grey and red boxes, respectively. Branch support values displaying branch support for the Ta-SETup proteins are indicated in the form ultra-fast bootstrap/alrt-sh like bootstrap values and were computed using iqtree software version 1.65.

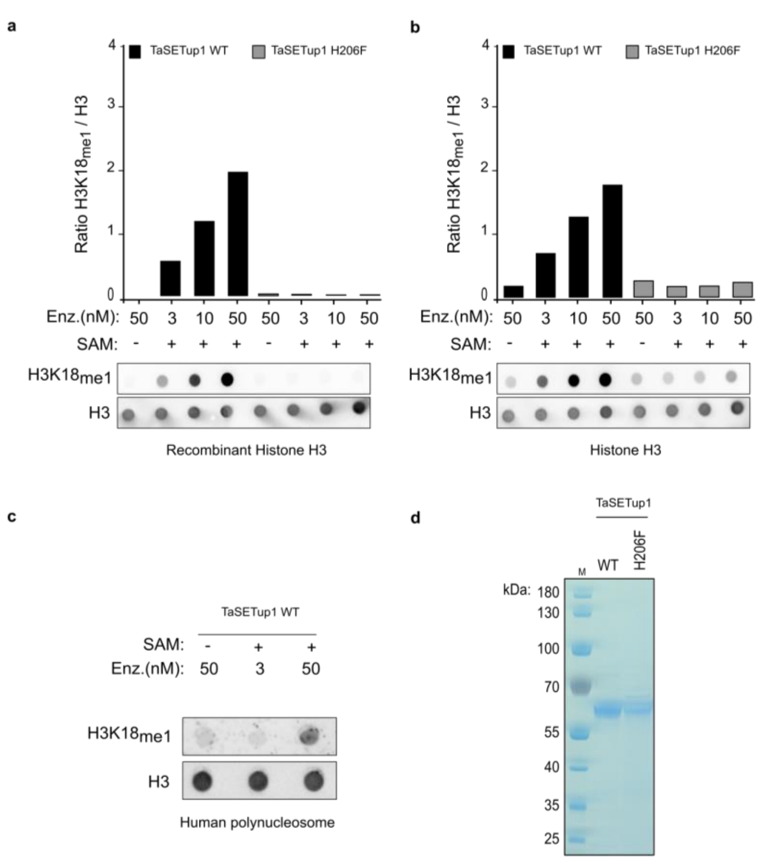

Supplementary Figure 12: Assays of methyltransferase activity *in vitro* for TaSETup1

a. Methyltransferase assay of recombinant TaSETup1 (wild-type WT or mutant H206F) incubated, with or without SAM and recombinant Histone H3, followed by immuno-dot-blot detection with a H3K18me1 antibody. Results were normalized to histone H3.

b. As above, methyltransferase assay of recombinant TaSETup1 (wild-type WT or mutant H206F) using histone H3 from calf thymus as the substrate.

c. Methyltransferase assay of recombinant TaSETup1 wild-type incubated with or without SAM and human polynucleosomes purified from HeLa cells (EpiCypher) followed by immuno-dot-blot detection with the H3K18me1 antibody.

d. Coomassie staining of recombinant TaSETup1 wild-type WT or mutant H206F.

All these experiments were performed three times independently with similar results; these data shown represent one of these three experiments.

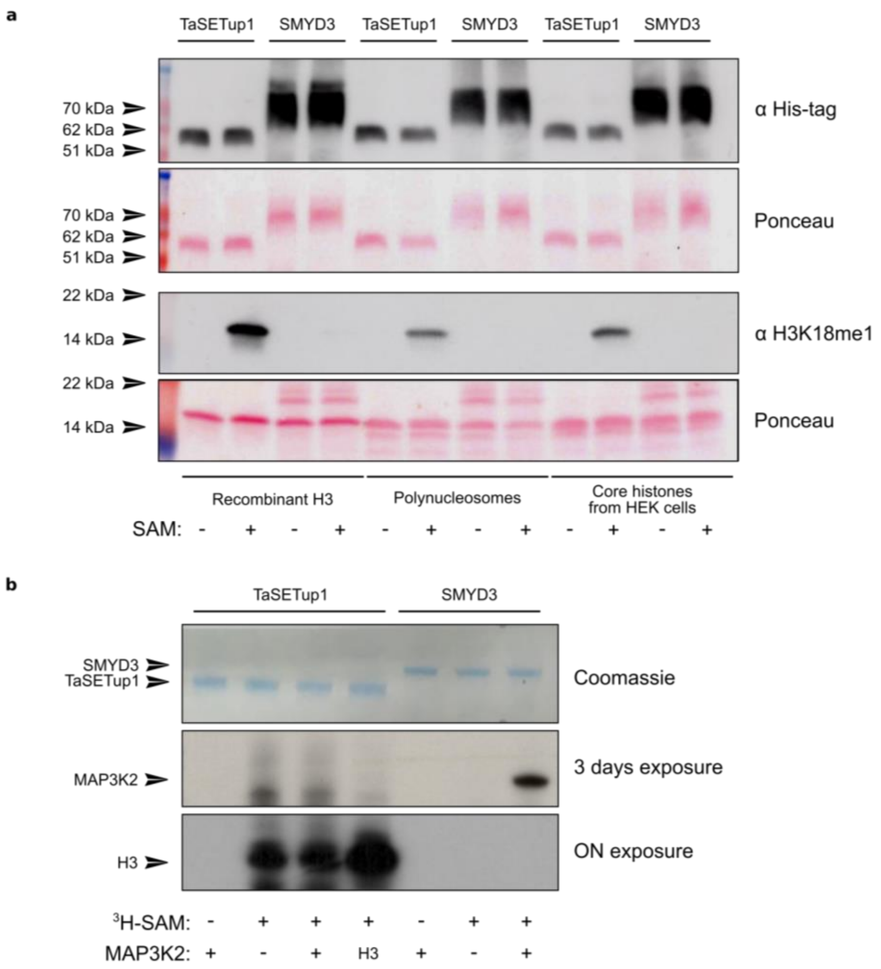

Supplementary Figure 13: Comparison of TaSETup1 and SMYD3 methyltransferase activity on different substrates

a. Methyltransferase assay of recombinant TaSETup1 or SMYD3 enzymes with recombinant histone H3, chicken polynucleosomes and core histones substrates extracted from HEK cells. 1 µg of enzyme was incubated with or without SAM and with histone substrates at 30°C overnight. Samples were then separated by SDS-PAGE followed by immuno-blot detection with an H3K18me1 antibody.

b. Radiometric methyltransferase assay of recombinant TaSETup1 or SMYD3 proteins with recombinant MAP3K2 or histone H3 substrates. 1 µg of enzyme was incubated with or without ^{3}H-SAM and with recombinant MAP3K2 or H3 substrate at 30°C overnight. Samples were separated by SDS-PAGE and gel was Coomassie stained. Incorporation of the ^{3}H-methyl group was detected as an autoradiographic signal at different time points.

All these experiments were performed three times independently with similar results; a representative of the three experiments is shown.

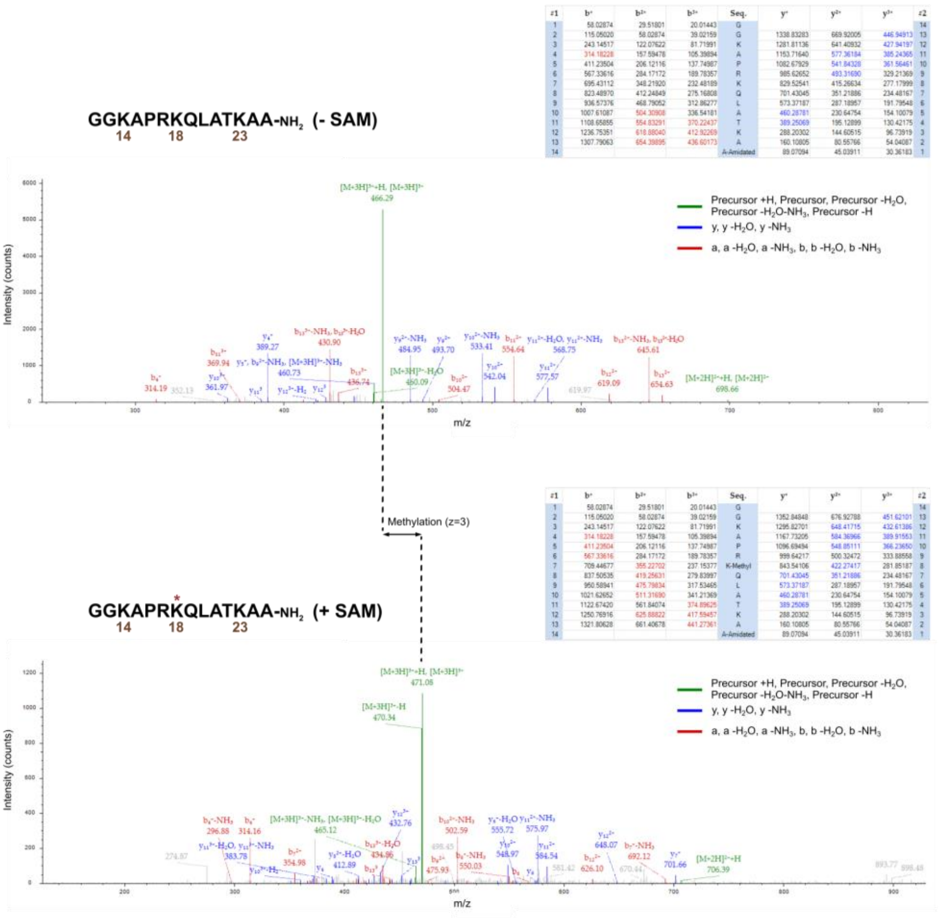

Supplementary Figure 14: Mass spectrometry characterization of H3K18 monomethylation by TaSETup1.

50 µg of a H3 derived 14-mer peptide flanking the lysine 18 were incubated with 3 µg TaSETup1 and with or without SAM for 2h at room temperature. Samples were analysed by LC-MS/MS as described in Methods. Upper panel shows the spectrum obtained in the sample without SAM, while the bottom panel displays the data obtained in the presence of SAM. The position of methylation is indicated on the H3K18 peptide sequence by an asterisk (*).

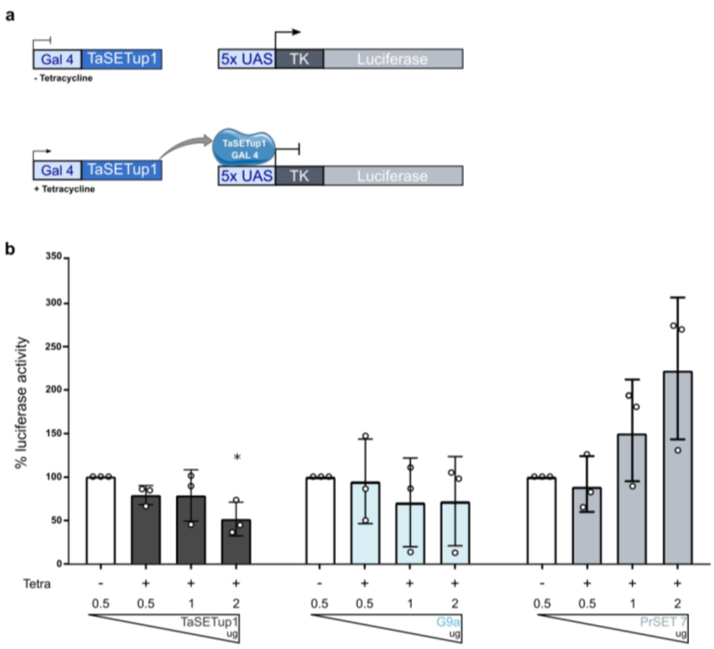

Supplementary Figure 15: Luciferase assay of TaSETup1 activity.

a. Schematic representation of 5X GAL4-UASTK-Luc reporter system, stably integrated in HEK293 cells (T-REx system). Cells were stably transfected with GAL4-TaSETup1 and grown in the presence or absence tetracycline for 48 h.

b. Repression of Luciferase expression with increasing amounts of GAL4-TaSETup1 compared to GAL4-G9a (known repressive KMT) or GAL4-PrSET7 (known activating KMT) in the presence or absence of tetracycline. Data are represented as percent of uninduced controls (mean ± SEM, n = 3).

Statistical Dunnett’s multiple comparison test, two-sided: ns=not statistically significant p>0,3976; *p<0.0299

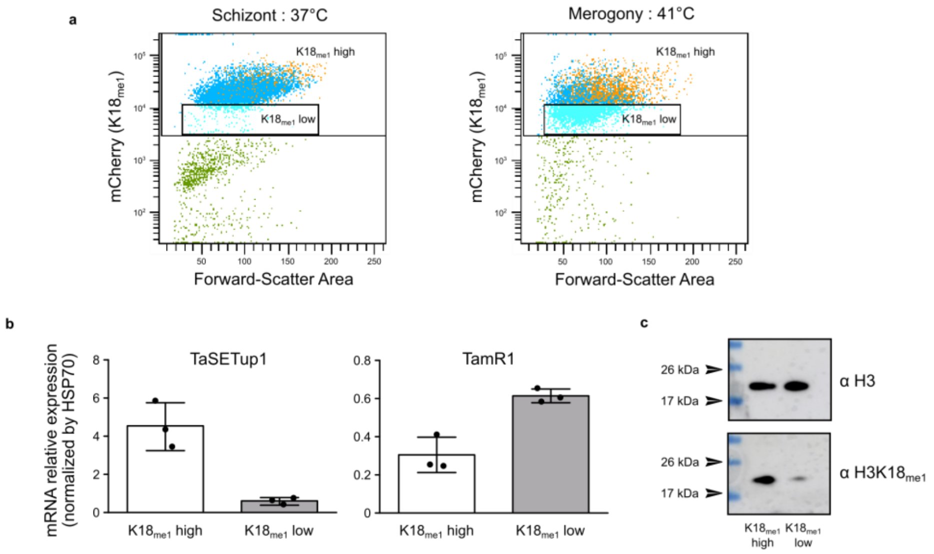

Supplementary Figure 16: Merogony is associated with reduced TaSETup1 and reduced H3K18me1.

a. Flow cytometry (FACS) analysis of Tac12 infected macrophage cells grown at 37°C (schizont stage, left panel) or incubated at 41°C (merogony stage, right panel). Live cells labeled with antibodies against H3K18me1 (mCherry) and sorted by forward scatter and mCherry intensity levels.

b. The two populations of cells (H3K18me1-hi and H3K18me1-lo) were analysed by RT-qPCR for the expression of the parasite *TamR1* gene (an indicator of merozoite differentiation) or *TaSETup1* (the schizont-expressed parasite KMT). The H3K18me-lo population was enriched for the TamR1 differentiation marker and showed reduced TaSETup1 expression. The graph shows the mean + SD for technical triplicates, n=3.

c. Western blot analysis of the two sorted cell populations with an antibody specific to H3K18me1. Immunoblotting for histone H3 was used as control. This experiment has been done in technical replicate n=3.

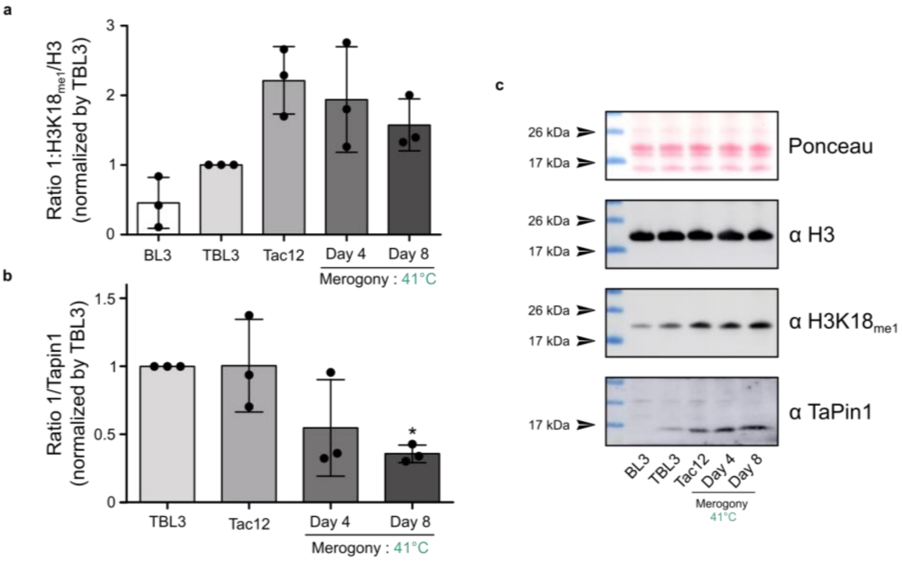

Supplementary Figure 17: Immunoblot analysis of cells undergoing merogony.

a. Graphical representation of the H3K18me1: Histone H3 ratio (the levels in TBL3 infected cells were set at 1) comparing BL3 and TBL3 cells, or TaC12 macrophages (in the schizont stage or at 2 merogony stages, day 4 or 8).

b. Graphic representation of the H3K18me1: Histone H3 ratio normalized for expression of TaPin1.

c. Western blot analysis of H3, H3K18me1 and the parasite Tapin1 protein in BL3, TBL3, or Tac12 cells (in the schizont stage or at 2 merogony stages, day 4 or 8)

These experiments were performed three times independently with similar results; these data show one of these three experiments. Full scans blots are included in the Source Data file.

Name	gene ID	Original annotation	Re-annotation (bl2go)	InterPro IDs
M2M1	TA21400	hypothetical protein	Tpr-related protein family member	no IPS match
M2M2	TA19275	hypothetical protein	hypothetical protein TA19275	IPR007480 (DUF529); SignalP
M2M3	TA05870	rhoptry-associated protein	rhoptry-associated protein	IPR004318 (Rhoptry-associated protein 1); SignalP
M2M4	TA17055	hypothetical protein	hypothetical protein TA17055	SignalP
M2M5	TA21080	Map2 kinase, putative	putative cell-cycle-associated protein kinase MAPK	IPR000719 (Protein kinase domain)
M2M6	TA18005	hypothetical protein	hypothetical protein TA18005	IPR022742(serine aminopeptidase, S33); IPR007480 (DUF529); IPR029058 (alpha/beta hydrolase fold)
M2M7	TA07305	hypothetical protein	hypothetical protein TA07305	no IPS match
M2M8	TA14210	hypothetical protein	membrane protein	SignalP; Tmmhelix
M2M9	TA19675	hypothetical protein	AT hook motif-containing protein	no IPS match
M2M10	TA07435	SfiI-subtelomeric fragment	SfiI-subtelomeric fragment related protein family member	IPR007480 (DUF529)
M2M11	TA14205	hypothetical protein	SfiI-subtelomeric fragment related protein family member	SignalP
M2M12	TA16155	hypothetical protein	DNA topoisomerase VI, b subunit	IPR036890 (Histidine kinase/HSP90-like ATPase)
M2M13	TA16660	hypothetical protein	rhoptry neck protein 5	Tmhelix
M2M14	TA13045	hypothetical protein	putative duplicated carbonic anhydrase	IPR001148 (Alpha carbonic anhydrase domain); SignalP
M2M15	TA16420	hypothetical protein	hypothetical protein TA16420	SignalP
M2M16	TA03755	sporozoite surface antigen	sporozoite surface protein p67	IPR008845 (Sporozoite P67 surface antigen); SignalP; Tmhelix
M2M17	TA08360	hypothetical protein	protein 82	IPR007480 (DUF529); SignalP
M2M18	TA21395	hypothetical protein	Tpr-related protein family member	Tmhelix
M2M19	TA04795	hypothetical protein	Tpr-related protein family member	SignalP
M2M20	TA04825	hypothetical protein	Tpr-related protein family member	SignalP; Tmhelix

Supplementary Table 1: 20 Cluster IV genes associated with life cycle transitions.

Original annotation (and functional re-annotation) of the 20 Cluster IV genes (i.e. enriched H3K18me1) that we identified as potentially up-regulated in macroschizont to merozoite transition (M2M), downregulated in sporozoite to macroschizont transition (S2M). The table shows the *Theileria* gene IDs, re-annotation derived from Blast2GO, official annotation available at PiroplasmaDB and domain content of the 20 genes (see Fig. 4b).

Gene ID	original annotat on	Re-annotat on (blast2go)	InterPro IDs
TA15445	hypothet cal protein	Tpr-related protein family member	Tmhelix
TA19610	Theileria-specif c hypothet cal telomeric sf i fragment-related protein	diacylglycerol O-acyltransferase	no IPS match
TA21205	hypothet cal protein	hypothet cal protein TA21205	SignalP
TA04355	hypothet cal protein	pentatricopept de repeat containing protein	no IPS match
TA18855	Tpr-related protein family member	Sf I-subtelomeric fragment related protein family member	DUF529 (IPR007480), SignalP
TA09600	cysteine proteinase	Tpr-related protein family member	Tmhelix
TA04130	hypothet cal protein	alpha/beta hydrolase	no IPS match
TA17491	hypothet cal protein	Sf I-subtelomeric fragment related protein family member	no IPS match
TA17325	Tpr-related protein family member	integral membrane protein	TMhelix
TA17115	Tpr-related protein family member	Sf I-subtelomeric fragment related protein family member	IPR007480 ;SignalP
TA08525	hypothet cal protein	inner membrane complex protein 1e	IPR022086
TA17100	hypothet cal protein	hypothet cal protein TA17100	no IPS match
TA07025	Tpr-related protein family member	Tpr-related protein family member	TMhelix
TA20150	hypothet cal protein	Tpr-related protein family member	TMhelix
TA14310	hypothet cal protein	ubiquit n-protein ligase	SignalP
TA07162	hypothet cal protein	hypothet cal protein TA07162	SignalP
TA19445	hypothet cal protein	rhoptry neck protein 2	TMhelix
TA14665	hypothet cal protein	Class II myosin heavy chain	SignalP; TMhelix
TA18780	hypothet cal protein	integrin beta-1-like isoform X2	no IPS match
TA05245	hypothet cal protein	Tpr-related protein family member	TMhelix
TA07920	Tpr-related protein family member	hypothet cal protein TA07920	no IPS match
TA21385	ubiquit n-conjugat ng enzyme E2	Tpr-related protein family member	no IPS match
TA13940	hypothet cal membrane protein	Tpr-related protein family member	TMhelix
TA21390	hypothet cal protein	Tpr-related protein family member	TMhelix
TA21380	phosphate transporter	Tpr-related protein family member	no IPS match
TA16050	hypothet cal protein	Sf I-subtelomeric fragment related protein family member	IPR007480 ; SignalP
TA11680	hypothet cal protein	perforin-like protein plp1	IPR020864 ; SignalP
TA16485	hypothet cal protein	transcript on factor with AP2 domain-containing protein	IPR028078 ; IPR001471
TA05615	Tpr-related protein family member	hypothet cal protein TA05615	SignalP
TA09115	Sf I-subtelomeric fragment related protein family member	membrane protein	TMhelix
TA07985	hypothet cal protein	hypothet cal product	IPR036259 ; TMhelix
TA10690	hypothet cal protein	ubiquit n-conjugat ng enzyme subfamily protein	IPR000608 ; IPR016135
TA13540	hypothet cal protein	cyclic nucleot de-binding domain containing protein	IPR000595 ; IPR018490 ; IPR018490 ; IPR011992
TA21050	hypothet cal protein	B-cell receptor CD22 isoform X1	TMhelix
TA18600	hypothet cal protein	integral membrane protein	TMhelix
TA18275	Tpr-related protein family member	early endosome ant gen 1	no IPS match
TA03521	Tpr-related protein family member	Sf I-subtelomeric fragment related protein family member	IPR007480
TA14955	hypothet cal protein	signal pept de containing protein	IPR007480 ; SignalP
TA17358	Tpr-related protein family member	Sf I-subtelomeric fragment related protein family member	IPR007480
TA20020	Sf I-subtelomeric fragment related protein family member	putat ve anonymous ant gen-1	IPR016024
TA18980	hypothet cal protein	putat ve transmembrane protein	no IPS match
TA21375	hypothet cal protein	Tpr-related protein family member	no IPS match
TA17490	Sf I-subtelomeric fragment related protein family member	Sf I-subtelomeric fragment related protein family member	no IPS match
TA13530	integral membrane protein	phosphate transporter	IPR001204 ; TMhelix
TA13535	Theileria-specif c hypothet cal telomeric sf i fragment-related protein	cyclic nucleot de-binding domain containing protein	IPR010875
TA04105	Sf I-fragment related sub-telomeric hypothet cal protein family member	dipept dyl aminopept dase 2	IPR000668 ; IPR014882 ;SignalP ; IPR036496 ; IPR038765
TA07585	Sf I-fragment related sub-telomeric hypothet cal protein family member	CTD nuclear envelope phosphatase 1	TMhelix
TA21370	Sf I-subtelomeric fragment related protein family member	putat ve transmembrane protein	IPR021366 ;TMhelix
TA13515	aspartyl(acid) protease	transcript on factor with AP2 domain(s)	IPR001471
TA15500	hypothet cal protein	Tpr-related protein family member	TMhelix
TA17685	hypothet cal protein	plasmepsin V	IPR033121 ;IPR021109
TA03855	hypothet cal protein	Tpr-related protein family member	TMhelix
TA03300	Sf I-subtelomeric fragment related protein family member	hypothet cal protein TA03300	IPR007480 ; SignalP
TA19115	hypothet cal protein	Sf I-subtelomeric fragment related protein family member	IPR007480
TA14680	Sf I-subtelomeric fragment related protein family member	hypothet cal protein TA14680	SignalP; TMhelix
TA06795	hypothet cal protein	Filamin/ABP280 repeat-containing protein	IPR017868 ; IPR014756
TA04790	hypothet cal protein	Tpr-related protein family member	TMhelix
TA03540	hypothet cal protein	hypothet cal protein TA03540	SignalP
TA15485	Theileria-specif c hypothet cal protein	Tpr-related protein family member	TMhelix
TA14120	hypothet cal protein	Tpr-related protein family member	no IPS match
TA20220	hypothet cal protein	200 kDa ant gen p200	no IPS match
TA07955	hypothet cal protein	hypothet cal protein TA07955	no IPS match
TA15095	hypothet cal protein	Tpr-related protein family member	no IPS match
TA14285	hypothet cal protein	Sf I-subtelomeric fragment related protein family member	IPR007480 ; SignalP
TA14835	hypothet cal protein	hypothet cal protein TA14835	no IPS match
TA17500	hypothet cal protein	Sf I-subtelomeric fragment related protein family member	no IPS match
TA03845	Theileria parva Tpr-related protein	Tpr-related protein family member	TMhelix

Supplementary Table 2: 67 Cluster IV genes associated with M2M transitions.

Original annotation (and functional re-annotation) of the 67 M2M_Cluster IV genes potentially up regulated in macroschizont to merozoite transition (M2M). The table shows the *Theileria* gene IDs, re-annotation derived from Blast2GO, official annotation available at PiroplasmaDB and domain content of the 67 genes (see Fig. 4b).

Gene ID	Protein ID	cluster	Description
TA18095	Q4UB32	1	
TA02615	Q4UD26	1	
TA16105	Q4UIG3	1	
TA16535	Q4UIY2	1	
TA17415	Q4UAU9	2	
TA12015	Q4UDT1	2	TaAP2.me2
TA09965	Q4U8Q5	3	
TA07550	Q4UA38	3	
TA04145	Q4UCX3	3	
TA11665	Q4UDL7	3	
TA20595	Q4UH38	3	
TA11145	Q4U8H9	4	TaAP2.me1
TA10940	Q4U982	4	
TA08375	Q4U9N8	4	
TA07100	Q4UAC2	4	
TA05055	Q4UBQ3	4	
TA13515	Q4UEK3	4	TaAP2.g
TA19920	Q4UG29	4	
TA06995	Q4UHX0	4	
TA16485	Q4UIX2	4	TaAP2.me3

Supplementary Table 3: List of *Theileria* ApiAP2 genes and their H3K18me1 status (cluster).

Table showing the GeneID, uniprotID and the cluster assignment for all the 20 *Theileria* ApiAP2 genes.

Name	Uniprot/interpro	piroplasmadb	ensembl	Gene_id	refseq_prot	refseq_mrna	Cluster #	gene size (nt)	# exons	prot size (aa)	mass (kDa)
TaSETup1	Q4UHT5	TA06820	CAI73354	3863755	XP_954031	XM_948938	II	1473	8	490	56,8
TaSETup2	Q4UGM0	TA21435	CAI3769	3863445	XP_954446	XM_949353	III	2532	7	843	97
TaSETup3	Q4U8N4	TA09850	CAI76819	3863308	XP_953444	XM_948351	IV	3252	2	1083	122,4
TaSETup4	Q4UBM9	TA05190	CAI75772	3864564	XP8955248	XM_950155	IV	4128	4	1375	159,5
TaSETup5	Q4U8P1	TA09890	CAI76812	3863102	XP_953439	XM_948344	IV	10788	9	3595	415,4
KDM1	Q4UG30	TA19925	CAI73959	3864155	XP_954636	XM_949543	III	2624	12	709	81,7
KDM2	Q4UEH4	TA13355	CAI74515	3861560	XP_952247	XM_947154	II	1181	5	324	37,1

Supplementary Table 4: List of *Theileria* SET-domain containing genes (*TaSETup* genes).
Table showing the gene references, cluster allocation and predicted protein size for the five SETup protein methyltransferases in the *T.annulata* genome (see Supplementary Fig. S10].

Target genes	Forward	Reverse
**Cloning**		
Gal4_SetB_SmaI_F / NotI_R	ATCCCGGGCATGTACAATAATCGCAATGAAGAAACG	TCGAGCGGCCGCTCATGCTTCATCAGGTGATTCT
TaSET-B_Gateway	ggggacaactttgtacaaaaaagttggcatgTACAATAATCGCAATGAAGAA	ggggacaactttgtacaagaaagttggttaTGCTTCATCAGGTGATTCTAC
TaSET-B H206F	CCTCATGCTGTTGGCATCACAC	GGTTACAGCTAAAGGCGACATAGG
**qPCR**		
TamR1	CCACTCCTGTAGCGGGTAAA	TTGTGGAGGTACTGACCCAAA
HSP70	ACGCAAATGGAATCCTCAAC	TATTCGTCGTGCTCTGCTAA
TaSETup4	TGGGAAACTATCACCCGCAC	GTCGCCAAACCAACAGCAAT
TaSETup5	ATCGAGACTTACGACGTGCC	ATGCTGTGCACCAGCTTACT
TaSETup3	TTGTCGGGGCAAAGTTGAGT	GTGGAAAACGCCGAGTATGC
TaSETup2	AAGTGAGCACACCCGATGTT	TGTACGGCTCAACTCCGAAC
TaSETup1	GATATTCGCAGAATCGCCGC	TGGTTTCGGTTTTCCACCCA
HDM1_TA19925	AGCGGACCTCAACGTCTTTT	TGAGCAGTGACCCATCACAC
HDM2_TA13355	TACGGCAATGAAGACCCAGC	CGCCTTCTGGAGATGAACCG
M1- TA21400	ACTAGGGCTGGTAATGGTGC	TGGAGCTTTGTCGTAGGCTT
M2- TA19275	AAGCCGGTGACACATACAGT	CCGGCATTTCGTAGGGGTTT
M3- TA05870	TGGTCCAGGTTAGTGTTGCG	GCCTAGCGAGCACCAGTATG
M4- TA17055	GCATCGTTGTTTGGACTGGC	TAGTCCTTGCGCACATCGTC
M5- TA21080	GCACTGGAACTGGTGCAAAA	TAGGACCCTGAGCCAACCAT
M6- TA18005	CAATGTGCGATGTCAACGGG	GATCGGCATCACATTCGGGA
M7- TA07305	TGAGAGTGCATCAGAGGACG	ACTCGTCCAACAAAGGCTCA
M8- TA14210	TGGGCAGAATGGGAATGGTC	ATCCTATCGCAACTCCTGCG
M9- TA19675	GGCAGAACAAGGGTTAGGGA	CTCTCGAGCGCATCATCATCT
M10- TA07435	AAAGAAGGGCCGATACCGTT	TGGAACCCATTGACCCAGTT
M11- TA14205	CGAGACCTGTTTCCGGTGAA	TCATCCCACGTTTTGGCAGT
M12- TA16155	TGCAATTGACGCATGCAAAGA	CCCGACCCATCATCGAAACA
M13- TA16660	GGCCGTGTCTTAGATGAGCA	CCTCCCTTTGGCGCATAGAT
M14- TA13045	TGGCATGTGTAGTGCTGGATT	ACCATGTCACCTCGTTTCCA
M15- TA16420	TCCGCATGTGAAGCAGTATCT	CTCGAACCTCCGGTAAGTTGT
M16- TA03755	GGAGGATCACTAAGGGGGCA	TGAGGGGTCTGACGGTACAT
M17- TA08360	ATTTTTCAAAGCGGGTGCCG	CGTGCTTTCCGTCCCTAACA
M18- TA21395	GGTAAAACATCCGATGGCGG	AGCGATGATGGTGCCAGTTA
M19- TA04795	CCAACGGCACTCTAAGTGGT	TCACCAGTGCCATCATCGAG
M20- TA04825	GCTAAAAGAAGCTGTCGGCG	TCTTACTTGGTGTCCACCGC

Supplementary Table 5: List of oligonucleotides used in this study for cloning, qPCR analysis or ChIP-PCR experiments.
