# Supplementary material for: Dynamic methylation of histone H3K18 in differentiating Theileria parasites
Source: Nat Commun. 2021 May 28;12:3221. doi: 10.1038/s41467-021-23477-2 (PMC8163883; doi:10.1038/s41467-021-23477-2)
Supplement: Supplementary file 2 — Reporting Summary [file 41467_2021_23477_MOESM2_ESM.pdf]

nature research

Corresponding author(s): Jonathan B. WEITZMAN

Last updated by author(s): Dec 21, 2020

Reporting Summary

Nature Research wishes to improve the reproducibility of the work that we publish. This form provides structure for consistency and transparency in reporting. For further information on Nature Research policies, see our Editorial Policies and the Editorial Policy Checklist.

Statistics

For all statistical analyses, confirm that the following items are present in the figure legend, table legend, main text, or Methods section.

n/a	Confirmed	
☐	☒	The exact sample size (*n*) for each experimental group/condition, given as a discrete number and unit of measurement
☐	☒	A statement on whether measurements were taken from distinct samples or whether the same sample was measured repeatedly
☐	☒	The statistical test(s) used AND whether they are one- or two-sided<br>*Only common tests should be described solely by name; describe more complex techniques in the Methods section.*
☐	☒	A description of all covariates tested
☐	☒	A description of any assumptions or corrections, such as tests of normality and adjustment for multiple comparisons
☐	☒	A full description of the statistical parameters including central tendency (e.g. means) or other basic estimates (e.g. regression coefficient) AND variation (e.g. standard deviation) or associated estimates of uncertainty (e.g. confidence intervals)
☐	☒	For null hypothesis testing, the test statistic (e.g. *F*, *t*, *r*) with confidence intervals, effect sizes, degrees of freedom and *P* value noted<br>*Give P values as exact values whenever suitable.*
☒	☐	For Bayesian analysis, information on the choice of priors and Markov chain Monte Carlo settings
☒	☐	For hierarchical and complex designs, identification of the appropriate level for tests and full reporting of outcomes
☒	☐	Estimates of effect sizes (e.g. Cohen's *d*, Pearson's *r*), indicating how they were calculated

Our web collection on statistics for biologists contains articles on many of the points above.

Software and code

Policy information about availability of computer code

Data collection	No software were used for data collection.
Data analysis	For RNA-Seq<br>Read-quality control was performed using fastQC (v0.11.7) and Rsubread38 (v1.26.1) qualityScore function. Read mapping was performed using the Rsubread align function on an indexed version of T. annulata genome assembly ASM322v1.32. RPKM values were obtained using the featureCounts function from the Rsubread package using the same ASM322v1.32 annotations. Visualisation and snapshots of bam files was performed using IGV (v2.3.91) and further modified using inkscape (v0.92.3). Kruskal-Wallis and pairwise Wilcoxon Statistical testing on RPKM values from cluster I-V was performed using the ggpubr (v0.2) R package.<br>ChIP-seq analysis is described below.<br>For image analysis:<br>We used ImageJ (NIH, version 1.53) and MetaMorph® Image Analysis Software (Molecular Devices®, version 7.10.2.240)<br>For the phylogeny and comparative genomics analysis we usedthe following software: mafft (v7.245), Jalview (v2.10.5),iqtree (v1.5.5) and FigTree (v1.4.3).

For manuscripts utilizing custom algorithms or software that are central to the research but not yet described in published literature, software must be made available to editors and reviewers. We strongly encourage code deposition in a community repository (e.g. GitHub). See the Nature Research guidelines for submitting code & software for further information.

Data

Policy information about availability of data

All manuscripts must include a data availability statement. This statement should provide the following information, where applicable:

- Accession codes, unique identifiers, or web links for publicly available datasets
- A list of figures that have associated raw data
- A description of any restrictions on data availability

All data generated or analyzed during this study are included in this published article. The RNA-Sequencing and ChIP-Seq data have been deposited to the ENA database with the study identifier #PRJEB3379. The Zenodo access link is provided (https://doi.org/10.5281/zenodo.3370034) DOI number : 10.5281/zenodo.3370034. Content description : This repository provides bigwig files for ChIP and RNA sequencing experiments in Theileria annulata. DeepTools generated Coverage files, ReadCount normalised files and SES normalised files are provided. Also provided are genome and annotation files for visualisation with IGV software. The Mass Spectrometry proteomics data were deposited in the ProteomeXchange Consortium via the PRIDE partner repository with the dataset identifier PXD024599. Source data are provided with this paper.

Field-specific reporting

Please select the one below that is the best fit for your research. If you are not sure, read the appropriate sections before making your selection.

☒ Life sciences ☐ Behavioural & social sciences ☐ Ecological, evolutionary & environmental sciences

For a reference copy of the document with all sections, see nature.com/documents/nr-reporting-summary-flat.pdf

Life sciences study design

All studies must disclose on these points even when the disclosure is negative.

Sample size	Sample size was determined to be adequate based on the magnitude and consistency of measurable differences between groups. Sample size decisions were based on the standards used in publications in the field. For all microscopy quantification, we sampled over 50 cells and performed all experiments in triplicate.
Data exclusions	Data were only excluded for failed experiments.
Replication	All experiments were replicated at distinct dates at least three times. Replicate experiments were successful.
Randomization	After discussion with colleagues it was decided that randomization approaches were not relevant for this study.
Blinding	We considered that blinding was not relevant for most of these experiments based on data reproducibility. An exception was the quantification of induction of merogony experiments, in which two independent researchers performed quantification under blinded conditions.

Reporting for specific materials, systems and methods

We require information from authors about some types of materials, experimental systems and methods used in many studies. Here, indicate whether each material, system or method listed is relevant to your study. If you are not sure if a list item applies to your research, read the appropriate section before selecting a response.

Materials & experimental systems

n/a	Involved in the study	
☐	☒	Antibodies
☐	☒	Eukaryotic cell lines
☒	☐	Palaeontology and archaeology
☒	☐	Animals and other organisms
☒	☐	Human research participants
☒	☐	Clinical data
☒	☐	Dual use research of concern

Methods

n/a	Involved in the study	
☐	☒	ChIP-seq
☒	☐	Flow cytometry
☒	☐	MRI-based neuroimaging

Antibodies

Antibodies used	We used these antibodies:<br>anti-H3K18me1 (ab177253, Abcam), 1:5000; anti-H3K18me1 (#31259, Active Motif), 1:10000; anti-H3K18me1 (#ab177253 Mellor's laboratory, Abcam) 1:10000; anti-H3K18ac (9675 S, Cell signaling) 1:800; anti-H3K18ac (ab1191, Abcam) 1:2000; anti-H3K4me3 (pAb-003-050, Diagenode) 1:200; anti-H3K4me3 (07-473, Millipore); anti-H3K36me3 (ab9050, Abcam); anti-H3 (ab1791, Abcam) 1:10000; Alexa594-conjugated donkey anti-rabbit antibody, 1:1000. (A32754, ThermoFischer); IgG isotype control (Cell Signaling,

27295); ProLong Gold Antifade Mountant with DAPI (Invitrogen)

anti-6xHis-tag (SAB270221, Sigma), 1:10000
anti-mouse HRP-secondary (G-2104, Thermofisher), 1:50000 (seulement pour les figures supp de type s3)
anti-rabbit HRP-secondary (31460, Thermofisher), 1:50000 (seulement pour les figures supp de type s3)

Validation

normal rabbit IgG (Cell Signaling, Ref : 2729S) : https://www.cellsignal.com/products/primary-antibodies
monoclonal anti-α-Tubulin (Sigma Aldrich, Ref : T9026) : https://www.sigmaaldrich.com

The three H3K18me1 antibodies were tested and validated in the experiments of the Figure S3; plus anti-H3K18me1 (ab177253, Abcam) https://www.abcam.com/histone-h3-mono-methyl-k18-antibody-epr17710-ab177253.html
H3K18ac (Cell signaling #9675) https://www.cellsignal.com/products/primary-antibodies/acetyl-histone-h3-lys18-antibody/9675
anti-H3K18ac (ab1191, Abcam) https://www.abcam.com/histone-h3-acetyl-k18-antibody-chip-grade-ab1191.html
anti-H3K4me3 (pAb-003-050, Diagenode). https://www.diagenode.com/en/p/h3k4me3-polyclonal-antibody-premium-50-ug-50-ul
anti-H3K4me3 (07-473, Millipore) https://www.merckmillipore.com/FR/fr/product/Anti-trimethyl-Histone-H3-Lys4-Antibody,MM_NF-07-473
anti-H3K36me3 (ab9050, Abcam) https://www.abcam.com/histone-h3-tri-methyl-k36-antibody-chip-grade-ab9050.html
anti-H3 (ab1791, Abcam) https://www.abcam.com/histone-h3-antibody-nuclear-marker-and-chip-grade-ab1791.html
Alexa594-conjugated donkey anti-rabbit antibody, (A32754, ThermoFischer) https://www.thermofisher.com/antibody/product/Donkey-anti-Rabbit-IgG-H-L-Highly-Cross-Adsorbed-Secondary-Antibody-Polyclonal/A32754

Eukaryotic cell lines

Policy information about cell lines

Cell line source(s)

All infected bovine cell lines used in this study were previously described: TBL3 cells were derived from in vitro infection of the spontaneous bovine-B lymphosarcoma cell line, BL3, with Hissar stock of T.annulata. The TpMD409 lymphocyte cell line are infected with T. parva. The TaC12 is a T. annulata infected bovine macrophage cell line. All parasite-infected cell lines were provided by colleagues in the field as indicated in the text. HEK293 T-REx cells were obtained from the lab of AIT SI ALI Slimane, they stably express the tetracycline repressor protein (Mozetta et al, Molecular Cell 2014).

Authentication

The cells were authenticated as infected by Theileria parasites with PCR of several parasite genes

Mycoplasma contamination

All cells lines were tested negatively for mycoplasma on a monthly basis

Commonly misidentified lines
(See ICLAC register)

No cell lines used are listed in the database of commonly misidentified cell lines.

ChIP-seq

Data deposition

☒ Confirm that both raw and final processed data have been deposited in a public database such as GEO.

☒ Confirm that you have deposited or provided access to graph files (e.g. BED files) for the called peaks.

Data access links
May remain private before publication.

- Raw read files have been uploaded to ENA database (accession numbers below).
- Bigwig files have been uploaded to the Zenodo repository, with restricted access for editors and reviewers:

https://zenodo.org/record/3370034?
token=eyJhbGciOiJIUzUxMiIsImV4cCI6MTYwMDAzNDM5OSwiaWF0IjoxNTk3Mzk3NzcyfQ.eyJkYXRhIjp7InJlY2lkIjozMzcwMDM0fSwiaWQiOjkyOTEsInJuZCI6IjBlNDBmYjQ3In0.unX3QiyBNQm4LbzvEpoZSfytqf1efiVOd2vUa2fs9IIF77kCkNUXxvrs7ic7aF6qnLgWSXVJMS6B6SUP0b9TFA#.XzZc6qfitPY

this link gives access to coverage, normalised files and genome files and annotations for upload to a local version of IGV genome browser.

Files in database submission

All read files were deposited to the EMBL-ENA archive :
accession numbers (will be made public upon acceptance) :
study primary accession study secondary accession sample experiment Run submission date status
PRJEB33792 ERP116612 ERS3637559 ERX3492383 ERR3470720 06-Aug-2019 Confidential
PRJEB33792 ERP116612 ERS3637558 ERX3492382 ERR3470719 06-Aug-2019 Confidential
PRJEB33792 ERP116612 ERS3637557 ERX3492381 ERR3470718 06-Aug-2019 Confidential
PRJEB33792 ERP116612 ERS3637556 ERX3492380 ERR3470717 06-Aug-2019 Confidential
PRJEB33792 ERP116612 ERS3637555 ERX3492379 ERR3470716 06-Aug-2019 Confidential
PRJEB33792 ERP116612 ERS3637554 ERX3492378 ERR3470715 06-Aug-2019 Confidential
PRJEB33792 ERP116612 ERS3637553 ERX3492377 ERR3470714 06-Aug-2019 Confidential
PRJEB33792 ERP116612 ERS3637552 ERX3492376 ERR3470713 06-Aug-2019 Confidential
PRJEB33792 ERP116612 ERS3637551 ERX3492375 ERR3470712 06-Aug-2019 Confidential
PRJEB33792 ERP116612 ERS3637550 ERX3492374 ERR3470711 06-Aug-2019 Confidential

PRJEB33792 ERP116612 ERS3637549 ERX3492373 ERR3470710 06-Aug-2019 Confidential
PRJEB33792 ERP116612 ERS3637548 ERX3492372 ERR3470709 06-Aug-2019 Confidential
PRJEB33792 ERP116612 ERS3637547 ERX3492371 ERR3470708 06-Aug-2019 Confidential
PRJEB33792 ERP116612 ERS3637546 ERX3492370 ERR3470707 06-Aug-2019 Confidential
PRJEB33792 ERP116612 ERS3637545 ERX3492369 ERR3470706 06-Aug-2019 Confidential

Bigwig files of processed data can be accessed using the Zenodo link provided. (doi : 10.5281/zenodo.3370034)

Genome browser session (e.g. UCSC)

Theileria annulata is not available as a species at UCSC. We have provided a Zenodo download link with all the relevant files for visualisation using the IGV genome browser.

Methodology

Replicates

Two biological replicates were used for ChIP experiment in this study. Biological replicates correlation was assessed using PCA and Spearman/Pearson correlation coefficient computation using deepTools (v3.1.1) bamCoverage, multiBamSummary and plotCorrelation tools.

Sequencing depth

Reads are all 75 bp long, single-end reads.
mapping stats :
name library size uniquely mapped reads Total mapped reads multi mapping reads
TBL3_1_Input 24'452'865 459'003 472'578 reads (1,9%) 13'575
TBL3_2_Input 31'492'345 865'497 891'063 reads (2,8%) 25'566
TBL3_Input_H3K36_2 26'056'479 971'305 999'254 reads (3,8%) 27'949
TBL3_Input_H3K36_1 53'920'290 944'560 973'121 reads (1,8%) 28'561
TBL3_1_H3K4_me3 38'998'902 7'801'027 7'902'032 reads (20,3%) 101'005
TBL3_2_H3K4_me3 55'632'141 12'677'213 12'851'400 reads (23,1%) 174'187
TBL3_1_H3K18_me1 41'748'547 5'155'793 5'285'486 reads (12,7%) 129'693
TBL3_2_H3K18_me1 29'543'230 4'838'799 4'966'524 reads (16,8%) 127'725
TBL3_1_H3K18_ac 7'215'658 467'750 474'769 reads (6,6%) 7'019
TBL3_2_H3K18_ac 34'571'877 2'878'572 2'923'353 reads (8,5%) 44'781
TBL3_1_H3K36me3 51'845'808 3'262'866 3'338'231 reads (6,4%) 75'365
TBL3_2_H3K36me3 25'160'921 3'820'804 3'912'043 reads (15,5%) 91'239

Antibodies

anti-H3K18me1 (Abcam 177253), anti-H3K18Ac (Cell Signaling 9675S), anti-H3K4me3 (Millipore 07-473), anti-H3K36me3 (ab9050) and IgG isotype control (Cell Signaling 27295).

Peak calling parameters

ChIP enrichment for each gene and upstream region was assessed by using DeepTools computeMatrix plotFingerprint and plotHeatmap functions.
Read mapping was performed using the Rsubread align function on an indexed version of Theileria annulata genome assembly ASM322v1.32. Biological replicates correlation was assessed using PCA and Spearman/Pearson correlation coefficient computation using deepTools (v3.1.1) bamCoverage, multiBamSummary and plotCorrelation tools.
Peak calling was only used to further confirm differences between H3K18me1 and H3K36me3. Peak calling was performed using macs2 using default parameters of callpeak and bdgdiff functions.

Data quality

Raw reads were converted to Fastq files and their quality assessed using Aozan (version 2.2.1). Read-quality control was performed using fastQC (v0.11.7) and Rsubread (v1.26.1) qualityScore function.
Biological replicates correlation was assessed using PCA and Spearman/Pearson correlation coefficient computation using deepTools (v3.1.1) bamCoverage, multiBamSummary and plotCorrelation tools.
ChIP quality was assessed using DeepTools plotFingerprint function.

Software

FastQC, Rsubread, IGV and DeepTools were used for ChIP-seq analysis.
K-means clustering on H3K18me1 ChIPseq analysis was performed using the computeMatrix and plotHeatmap tools. Circos plot of ChIP-seq and RNA-seq experiment was done using circlize41 (v0.4.5).
